# The Canadian Women’s Heart Health Alliance ATLAS on the Epidemiology, Diagnosis, and Management of Cardiovascular Disease in Women — Chapter 8: Knowledge Gaps and Status of Existing Research Programs in Canada

**DOI:** 10.1016/j.cjco.2023.11.013

**Published:** 2023-11-17

**Authors:** Marie-Annick Clavel, Harriette G.C. Van Spall, Laura E. Mantella, Heather Foulds, Varinder Randhawa, Monica Parry, Kiera Liblik, Amy A. Kirkham, Lisa Cotie, Shahin Jaffer, Jill Bruneau, Tracey J.F. Colella, Sofia Ahmed, Abida Dhukai, Zoya Gomes, Najah Adreak, Lisa Keeping-Burke, Jayneel Limbachia, Shuangbo Liu, Karen E. Jacques, Kerri A. Mullen, Sharon L. Mulvagh, Colleen M. Norris

**Affiliations:** a Institut Universitaire de Cardiologie et de Pneumologie de Québec, Université Laval, Québec, Québec, Canada; b Department of Medicine, Department of Health Research Methods, Evidence, and Impact, McMaster University, Toronto, Ontario, Canada; c Department of Biomedical and Molecular Sciences, Queen’s University, Kingston, Ontario, Canada; d College of Kinesiology, University of Saskatchewan, Saskatoon, Saskatchewan, Canada; e Sunnybrook Health Sciences Centre, University of Toronto, Toronto, Ontario, Canada; f Lawrence S. Bloomberg Faculty of Nursing, University of Toronto, Toronto, Ontario, Canada; g Department of Medicine, Kingston Health Science Center, Kingston, Ontario, Canada; h Faculty of Kinesiology and Physical Education, University of Toronto, Toronto, Ontario, Canada; i Toronto Rehabilitation Institute (KITE), University Health Network, Toronto, Ontario, Canada; j General Internal Medicine, University of British Columbia, Vancouver, British Columbia, Canada; k Faculty of Nursing, Memorial University of Newfoundland and Labrador, St John, Newfoundland and Labrador, Canada; l Cumming School of Medicine, University of Calgary, Calgary, Alberta, Canada; m Faculty of Medicine, Division of Cardiology, Dalhousie University, Halifax, Nova Scotia, Canada; n Department of Surgery, University of British Columbia, Vancouver, British Columbia, Canada; o Department of Nursing and Health Sciences, University of New Brunswick, Saint John, New Brunswick, Canada; p Schulich School of Medicine, Western University, London, Ontario, Canada; q Section of Cardiology, Department of Medicine, Max Rady College of Medicine, University of Manitoba, Winnipeg, Manitoba, Canada; r Person with lived experience, Canadian Women’s Heart Health Alliance, Ottawa, Ontario, Canada; s University of Ottawa Heart Institute, Ottawa, Ontario, Canada; t Faculty of Nursing, University of Alberta, Edmonton, Alberta, Canada

## Abstract

Despite significant progress in medical research and public health efforts, gaps in knowledge of women’s heart health remain across epidemiology, presentation, management, outcomes, education, research, and publications. Historically, heart disease was viewed primarily as a condition in men and male individuals, leading to limited understanding of the unique risks and symptoms that women experience. These knowledge gaps are particularly problematic because globally heart disease is the leading cause of death for women. Until recently, sex and gender have not been addressed in cardiovascular research, including in preclinical and clinical research. Recruitment was often limited to male participants and individuals identifying as men, and data analysis according to sex or gender was not conducted, leading to a lack of data on how treatments and interventions might affect female patients and individuals who identify as women differently. This lack of data has led to suboptimal treatment and limitations in our understanding of the underlying mechanisms of heart disease in women, and is directly related to limited awareness and knowledge gaps in professional training and public education. Women are often unaware of their risk factors for heart disease or symptoms they might experience, leading to delays in diagnosis and treatments. Additionally, health care providers might not receive adequate training to diagnose and treat heart disease in women, leading to misdiagnosis or undertreatment. Addressing these knowledge gaps requires a multipronged approach, including education and policy change, built on evidence-based research. In this chapter we review the current state of existing cardiovascular research in Canada with a specific focus on women.

## Résumé

En dépit des avancées importantes de la recherche médicale et des efforts en santé publique, il reste des lacunes dans les connaissances sur la santé cardiaque des femmes sur les plans de l’épidémiologie, du tableau clinique, de la prise en charge, des résultats, de l’éducation, de la recherche et des publications. Du point de vue historique, la cardiopathie a d’abord été perçue comme une maladie qui touchait les hommes et les individus de sexe masculin. De ce fait, la compréhension des risques particuliers et des symptômes qu’éprouvent les femmes est limitée. Ces lacunes dans les connaissances posent particulièrement problème puisqu’à l’échelle mondiale la cardiopathie est la cause principale de décès chez les femmes. Jusqu’à récemment, la recherche en cardiologie, notamment la recherche préclinique et clinique, ne portait pas sur le sexe et le genre. Le recrutement souvent limité aux participants masculins et aux individus dont l’identité de genre correspond au sexe masculin et l’absence d’analyses de données en fonction du sexe ou du genre ont eu pour conséquence un manque de données sur la façon dont les traitements et les interventions nuisent aux patientes féminines et aux individus dont l’identité de genre correspond au sexe féminin, et ce, de façon différente. Cette absence de données a mené à un traitement sous-optimal et à des limites de notre compréhension des mécanismes sous-jacents de la cardiopathie chez les femmes, et est directement reliée à nos connaissances limitées, et à nos lacunes en formation professionnelle et en éducation du public. Le fait que les femmes ne connaissent souvent pas leurs facteurs de risque de maladies du cœur ou les symptômes qu’elles peuvent éprouver entraîne des retards de diagnostic et de traitements. De plus, le fait que les prestataires de soins de santé ne reçoivent pas la formation adéquate pour poser le diagnostic et traiter la cardiopathie chez les femmes les mène à poser un mauvais diagnostic ou à ne pas traiter suffisamment. Pour pallier ces lacunes de connaissances, il faut une approche à plusieurs volets, qui porte notamment sur l’éducation et les changements dans les politiques, et qui repose sur la recherche fondée sur des données probantes. Dans ce chapitre, nous passons en revue l’état actuel de la recherche existante sur les maladies cardiovasculaires au Canada, plus particulièrement chez les femmes.

***Lay Summary***

*Despite progress in medical research, a significant gap exists in knowledge of women’s heart health: many women are unaware of risk factors or symptoms, and health care providers often lack the appropriate relevant expertise. Clinical trials have focused predominantly on men/male participants, limiting our understanding of how cardiovascular treatments affect women. Prioritization of women’s heart health in research is required to address these gaps and provide a foundation for systemic change in education and public health policies.*

Despite sex and gender differences in pathophysiology, risk factors, presentation, drug metabolism, and treatment effects across cardiovascular (CV) conditions, women and female participants remain under-represented in research. As a result, decisions used to inform female and women’s care are often generalized from studies conducted primarily in men and male participants.^1^, ^2^, ^3^, ^4^, ^5^ Of note, female and male are used in this review when referring to sex particularities, whereas the use of men and women are associated with gender.

Regulatory and funding agencies have highlighted the importance of adequate inclusion of women and female participants in research studies, and have implemented policies requiring sex- and gender-specific analyses but little progress has been made in this regard.^4^^,^^6^ There remain large knowledge gaps in (1) unique CV conditions that affecting only female individuals (2) CV conditions that affect primarily women and female individuals; (3) CV treatments in pregnant or lactating women; and (4) CV conditions that present, progress, or respond to treatment differently in women and female patients. Individuals who have the potential to become pregnant or who are pregnant or lactating remain largely excluded from research because of outdated protection by exclusion ideologies.^5^ The net result is that there are limited safety and efficacy data to inform treatment decisions in female individuals and those who identify as women. The role of gender in health and disease is largely unknown, although is being increasingly explored. Women living with CV disease (CVD) in Canada are generally older than men, face related problems such as cognitive dysfunction and frailty, experience health care disparities, might have poorer quality of life, and might benefit more than men from certain health care resources.^7^^,^^8^ Large gaps in knowledge exist most acutely for transgender individuals, who are marginalized in clinical care and might be at higher CV risk and have poorer CV outcomes due to social, as well as biological factors such as gender-affirming hormone therapy. Closing the gaps in knowledge might facilitate better care as well as the development of sex- and gender-specific guidelines for women and gender-diverse individuals living with CVD.

**Figure 1:**
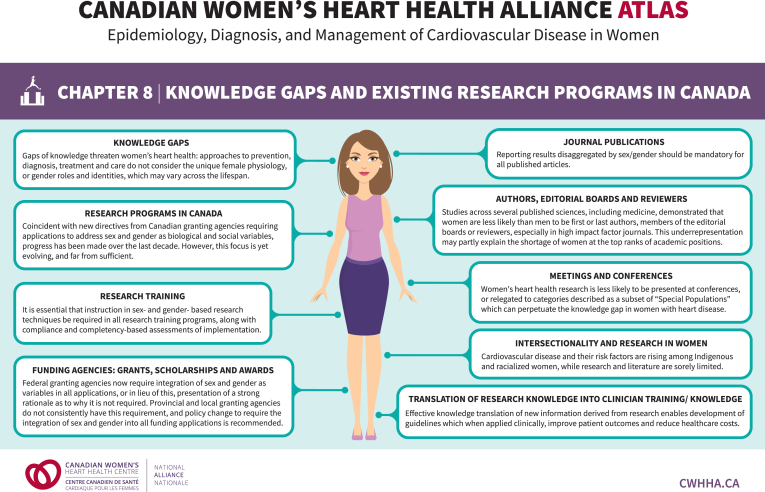
Summary of knowledge gaps and existing research programs in Canada. Modified with permission from the Canadian Women’s Heart Health Alliance.

Gaps in sex- and gender-specific knowledge, care, and research representation are pervasive and could be circumvented through dedicated women- and female-led training programs, clinical centres, and research networks. Indeed, research led by women focuses more on women’s CV health, recruits more women and ethnically diverse participants, and builds greater capacity for research expertise and leadership among women.^4^^,^^9^, ^10^, ^11^ The landscape for such training programs, services, and networks in Canada is currently unknown. The aim of this review is to define knowledge gaps, map existing resources, and identify areas for growth opportunities in women’s CV research in Canada. Figure 1 summarizes this chapter’s key concepts.

## Knowledge Gaps

***Gaps of knowledge threaten women’s heart health: approaches to prevention, diagnosis, treatment, and care do not consider the unique female physiology, or gender roles and identities, which might vary across the life span.***

Hormonal and other biological differences between male and female individuals require adequate representation of both sexes in cardiology research. There is a misconception that CVD occurs almost exclusively in men, likely driven by the exclusion of female representation in many studies and historically driven by a desire to reduce variability within research. From cell cultures that are most often derived from cells from male patients, to preclinical studies using primarily male animal models, the female sex is highly under-represented in CV research. Even when included in clinical trials, at best only approximately one-third of participants are women.^4^ Therefore, major knowledge gaps continue to exist.

Despite a net improvement since the early 1990s, propelled primarily by federal mandates in the United States of America, major research in women’s CV health is still lagging and urgently requires additional support to achieve parity. Risk factors for CVD are modulated by sex and gender. Indeed, untreated stage 1 hypertension causes more endothelial dysfunction in female compared with male patients.^12^ The effect of smoking is 2 times higher in women (even greater in younger women) than in men.^13^ Hypertriglyceridemia and diabetes are associated with worse outcomes in women compared with that in men.^14^ Unfortunately, the pathophysiological understanding of many of these differences is still lacking. Similarly, disorders occurring with sex-unique physiologic states, such as pregnancy, polycystic ovarian disease, and menopause, have been observed to be associated with increased CV risk, but they are under-researched and mechanisms have not yet been elucidated.

Despite well-known sex differences in cardiac remodelling,^15^^,^^16^ the sex-specific pathophysiology of CVD is not adequately explained. Examples include but are not limited to acute coronary syndrome (ACS) with nonobstructive coronary arteries, spontaneous coronary artery dissection, stress-induced cardiomyopathy, left ventricular remodelling, fibrosis deposition in patients with heart failure with preserved ejection fraction, and valvular heart disease.^2^^,^^17^, ^18^, ^19^

It is well known that compared with men, women with ACS have delayed presentation and referral.^20^^,^^21^ The reasons for this delay are thought to be multifactorial and associated with sex and gender differences, including symptom presentation, social roles, caregiving roles, and failure to prioritize self-care.^22^, ^23^, ^24^ Unfortunately, sex and gender effects on CVD diagnosis and prognosis are not yet fully understood.

Moreover, we have only recently become aware that cardiac biomarkers such as troponin and natriuretic peptides used in the diagnosis of cardiac disease have sex-specific thresholds for normative values, which are not consistently implemented.^25^^,^^26^ The underlying physiological explanations for these differences are unknown but might be related to differences in how female hearts respond to pressure and volume. Higher rates of atypical causes of ACS have been observed in women requiring assessment of specific biomarkers, which are rarely drawn. For example, in a female patient with an allergic history who presents with ACS and is shown to have no evidence of obstructive coronary artery disease (CAD), it is imperative for the tryptase enzyme to be checked promptly for the evaluation of allergic/anaphylactic coronary (Kounis) syndrome.^27^ Unfortunately, this enzyme is rarely drawn, or is drawn too late to be valid.

Although it has been observed that noninvasive and invasive diagnostic testing might have sex-specific variation in accuracy,^19^ no diagnostic strategy has been tested specifically in women.

Similarly, outcomes after procedural therapies have been shown to be different according to sex; women have worse outcomes than men after CV surgical interventions such as coronary artery bypass and valve replacement surgery but equivalent or better outcomes after percutaneous interventions.^28^, ^29^, ^30^ These variations are not fully understood, and results remain controversial. Medical therapies have been studied almost exclusively in men. Thus, the efficacy and adverse events specific to women are not clearly defined. Moreover, even when therapy has been proven effective and safe in women, it will be used less or at suboptimal implementation compared with that used in men. Fortunately, over the past decade, focused research programs in Canada have begun to emerge to address such knowledge gaps.

## Research Programs in Canada

***Coincident with new directives from Canadian*** ***granting*** ***agencies requiring applications to address sex and gender as biological and social variables, progress has been made over the past decade in the development of research studies and programs dedicated to women’s heart health. However, this focus is yet evolving, and far from sufficient. Moreover, sex- and gender-based analysis and reporting are yet to become mandatory for all research published in journals.***

Canada is a leader in CV research and is home to many expert scientists and clinicians studying CVD and related areas of health. Women-specific heart health research has been emerging over the past few decades, as evidenced by the establishment of focused programs within Canadian academic institutions and clinical centres of excellence. To evaluate the extent of women’s heart health research across Canada, the Canadian Women’s Heart Health Alliance (CWHHA) distributed a brief survey titled: “National survey: knowledge gaps and status of existing cardiac and/or stroke-focused research programs that incorporate sex and gender in Canada” to assess the depth, breadth, and current status of this research.

The survey was created by the CWHHA Knowledge Translation and Mobilization Working Group using the Research Electronic Data Capture (REDCap) database and distributed electronically in April 2022 using the CWHHA, the Canadian Institute of Health Research (CIHR), the Heart and Stroke Foundation of Canada (HSFC), and Canadian universities e-mail distribution lists. A total of 109 responses, representing the Canadian provinces of British Columbia, Alberta, Manitoba, Ontario, Quebec, Nova Scotia, and Newfoundland and Labrador, were received. Response results were reviewed and categorized according to the scientific area of research, study/laboratory name, primary investigator, and institution. Although not an exhaustive list of current programs of research, these results provided a snapshot of current CV research in women in Canada (Table 1). The results of this survey highlight the gaps in geographical representation and areas of research currently being conducted, thereby creating opportunities for future research directions, knowledge translation strategies, and collaborations to further develop and strengthen women’s heart health research initiatives across Canada.

**Table 1:** Nonexhaustive list of sex-specific research studies and programs ongoing in Canada, with principal investigators and institutions

Research topic/category	Project title or laboratory name	Principal investigator	Institution/faculty
Atrial fibrillation/thromboembolism	Sex differences in the risk of recurrent venous thromboembolism: testing an epigenetic hypothesis	France Gagnon	University of Toronto

Behavioural/psychological	SCAD psychosocial impacts	Heather Tulloch	University of Ottawa Heart Institute
	Cardiovascular Health Psychology and Behavioural Medicine Laboratory		
	Comparing the Effects of Cognitive Training and Physical Exercise on Cognition, Cerebral Autoregulation and Cerebral Vasoreactivity in Men and Women With Heart Failure (ReCARDIO)	Louis Bherer	Montreal Heart Institute
	**F**emale **R**isk Factors for Post-**I**nfarction **D**epression and **A**nxiety (FRIDA): Pilot Study	Kiera Liblik and Amer Johri	Queen’s University

Brain/cerebrovascular	Brain and Heart Nexus Research Program	Jodi Edwards	University of Ottawa Heart Institute
Cancer/oncology	Cardiometabolic, Oncology, Diet and Exercise for Women (CODE-W) research centre	Amy Kirkham	University of Toronto
	Edmonton multi-disciplinary Research (ENCORE) Group Edmonton Cardio Oncology	Edith Pituskin, Ian Paterson	University of Alberta
	Cardiotoxicity Prevention Program	Paaladinesh Thavendiranathan	University of Toronto

Coronary artery diseases	Myocardial infarction within 30 days of discharge: a descriptive study of Albertans	Colleen Norris	University of Alberta
	Prevalence and Long-term Impact of Nonatherosclerotic CAD (PRYME)	Jacqueline Saw	Vancouver Coastal Health/University of British Columbia
	CODE-MI (Cardiovascular Health at Centre for Health Evaluation and Outcome Sciences [CHEOS])	Karin Humphries	Vancouver Coastal Health
	hs-**c**Tn—**O**ptimizing the **D**iagnosis of Acut**e M**yocardial **I**nfarction/Injury in Women (CODE-MI)	Mona Izadnegahdar, Salima Jutha	University of British Columbia

Diagnostic and screening	Development and testing of the Cardiovascular Assessment Screening Program (CASP)	Jill Bruneau	Memorial University
Endocrine	Young women’s heart events and the association with female hormones	Colleen Norris	University of Alberta
Heart failure	Biological sex, sex chromosomes, and heart failure	Jacques Couet	Université Laval
	Cardiocore Big Data Research Unit	Louise Sun	University of Ottawa Heart Institute
	Sex differences in left ventricular function and frailty on cardiovascular structures	Susan Howlett	Dalhousie University

Pregnancy/menopause	What are the experiences of women who had gestational diabetes and are at risk for or go on to develop coronary artery disease?	April Pike	Memorial University
	Mobile health apps for pre-eclampsia	Emily Seto	University of Toronto
	Program for Pregnancy and Postpartum Health (PPPH)	Margie Davenport	University of Alberta
	Women’s maternal and perinatal cardiovascular health	Sandra Davidge	University of Alberta
	Women at the intersection of pregnancy and cardiovascular disease: the WRISQ cohort	Nathalie Auger	McGill University
Primary prevention/rehabilitation	Exercise Physiology and Cardiovascular Health Laboratory	Jennifer Reed	University of Ottawa Heart Institute
	Canadian Women’s Heart Health Centre	Kerri-Anne Mullen, Thais Coutinho	University of Ottawa Heart Institute
	Determining optimal exercise strategies and promoting a continuum of care for people after stroke Sex and gender differences and predictors of physical activity from the stroke event to the community Sex Differences in predictors of completion of a 6-month exercise-based cardiovascular rehabilitation program in 1536 people after stroke Sex differences in post-stroke depressive symptoms at entry to cardiac rehabilitation: a retrospective study	Susan Marzolini	University Health Network/Toronto Rehabilitation
	Why are women predisposed to greater depressive symptoms? A sex-, age-, and diagnosis-matched cardiac rehabilitation cohort	Susan Marzolini, Tracey Colella	University Health Network/Toronto Rehabilitation
	**W**omen’s **A**dvanced **R**isk Assessment in **M**anitoba (WARM)	Todd Duhamel	University of Manitoba
	Sex differences in predictors of completion of a 6-month adapted cardiac rehabilitation program	Paul Oh	University Health Network/Toronto Rehabilitation

Risk factors	**S**outh **A**sian Women **T**ogether in a **H**ealth **I**nitiative (SATHI): A Pilot Randomized Controlled Trial	Abida Dhukai	University of Toronto
	Sex differences in cardiac electrophysiology	Celine Fiset	Montreal Heart Institute
	Sex and gender factors associated with CVD	Colleen Norris	University of Alberta
	Heart health in high-risk women with and without PCOS Health outcomes in PCOS: focus on CVD and T2D risk Cardiac function and dyslipidemia in a PCOS-prone rodent model Fish oil to reduce atherogenic apob-remnant dyslipidemia and subclinical ACVD in high-risk women with and without PCOS	Donna Vine	University of Alberta
	Risk Evaluation and Stratification of Low Risk for Cardiovascular Disease in Women (RESOLVE)	Elsie Nguyen	University of Toronto
	Sex differences in cardiopulmonary diseases	Ketul Chaudhary	Dalhousie University
	Sex differences in arterial stiffness	Thais Coutinho	University of Ottawa Heart Institute

SCAD	**Can**adian **S**pontaneous **C**oronary **A**rtery **D**issection **(**CANSCAD) Registry	Jacqueline Saw	Vancouver Coastal Health/University of British Columbia
	Canadian spontaneous coronary artery dissection genetic study	John Mancini, Karin Humphries, Jacqueline Saw	Vancouver General Hospital/Vancouver Coastal Health/University of British Columbia

Systemic patient care	Women’s patient-centred care	Anna Gagliardi	University of Toronto
	Improving women’s heart health focusing on health services, as well as the effect of social, cultural, and environmental factors	Husam Abdel-Qadir	Women’s College Hospital
	Women’s narrative when attending the ER for heart-related symptomatology	Colleen Norris	University of Alberta
	Exploring Methods to Improve Participations Of Women in Clinical Trials to Help Enhance Stroke Recovery Research (EMPOW-HER)	Mark Bayley	University Health Network/Toronto Rehabilitation Institute
	Optimizing post-acute cardiovascular care globally	Sherry Grace	York University/University Health Network/Toronto Rehabilitation Institute
	Patient and caregiver experience	Susan Law	University of Toronto
	Seamless transitions in cardiac patient care	Tracey Colella	University Health Network/Toronto Rehabilitation Institute
	Cardiac Care and Determinants of Health - Decision Tools	Kathrine King Shear	University of Calgary
	Women’s heart health clinics in Canada; an assessment of demographics, clinical characteristics, and quality indictors	Sharon Mulvagh	Dalhousie University
Technology	Using machine learning to introduce more equitable representation of myocardial infarction images	Janessa Griffith	University of Toronto
	Development and Usability Testing of HEARTPA♀N: An Integrated Smartphone and Web-Based Intervention for Women With Cardiac Pain	Monica Parry	University of Toronto

Valvular heart diseases	Canada Research Chair in Women’s Cardiac Valvular Health Sex-dependent differences in pathophysiological mechanisms, presentation, outcome, and treatment of aortic valve stenosis Sex differences in presentation and outcomes of mitral regurgitation	Marie-Annick Clavel	Université Laval
Other research topic	Women’s cardiovascular health initiative	Sofia Ahmed	University of Calgary
	Circadian medicine and cardiovascular health Centre for Cardiovascular Investigations Southern Ontario Cardiovascular Research Association	Tami Martino	University of Guelph
	Effect of female sex hormones on heart function post-MI and heart failure	W. Glen Pyle	University of Guelph

ACVD, atherosclerotic cardiovascular disease; CVD, cardiovascular disease; ER, emergency room; MI, myocardial infarction; PCOS, polycystic ovary syndrome; SCAD, spontaneous coronary artery dissection; T2D, type 2 diabetes mellitus.

These findings support the need to amplify the number of research programs and studies focused on women’s heart health while simultaneously training future generations of scientists and clinicians promoting the use of a sex and gender lens.

## Research Training

***It is essential that instruction in sex- and gender-based research techniques be required in all research training programs, along with compliance and competency-based assessments of implementation.***

Recent mandates issued from our major Canadian sources for research funding, CIHR and HSFC, require that applications include sex and gender considerations in research design, data analysis, and reporting. Thus, it is essential that research trainees receive education to implement these skills early in their careers. Principal investigators (PIs) and research supervisors play a critical role in the development of our next generation of scientists and clinicians, to ensure that appropriate methods are used to address sex and gender considerations in every study and in every research subject.

In the CWHHA National Research Survey (distributed electronically in April 2022 and representing the Canadian provinces of British Columbia, Alberta, Manitoba, Ontario, Quebec, Nova Scotia, and Newfoundland and Labrador), we explored the integration of sex and gender training into existing research training programs through questions directed to current postdoctoral students as well as those in MSc or PhD programs. According to our results, 18 (35%) of 51 trainee respondents reported that their academic or research institution included sex and gender content in their course content, which was delivered by 32 different described methods. The most common methods of sex and gender research training for trainees in descending order of implementation were: (1) mandatory completion of the CIHR sex and gender module (https://cihr-irsc.gc.ca/e/50836.html); (2) unique training provided by individual program supervisors; (3) standardized course program content; and (4) institution-driven internal training (eg, Women’s College Hospital [Toronto, Ontario] “sex-specific analysis and reporting in clinical trials” online training module [http://womensxchange.womensresearch.ca/assets/emodules/SexSpecific/story_html5.html]).

Very few respondents reported that their institution had requirements to integrate sex and gender considerations into theses or dissertations (n = 4; 8%). One respondent noted that trainees were assessed on sex and gender integration at every committee meeting and their thesis defense.

The reported barriers to the implementation of policies requiring integration of sex and gender into research training included departmental, faculty, and/or institutional lack of necessary time (n = 8), lack of funding (n = 7), paucity of trained research faculty (n = 6), lack of perception regarding the requirement for funding success (n = 5), inadequate resources for local research team (n = 5), and disinterest or disinclination by researchers (n = 4). Specific to requirements to consider sex and gender in theses and dissertations, one specific barrier mentioned was that dissertation formatting requirements are instituted centrally by the University, not within individual programs. Although such policies are critical for ensuring inclusion, diversity, equity, and access in health-related and biomedical research in cells, animals, or humans, they would not apply to several other research disciplines and graduate programs.

We recommend that, at a minimum, all health research trainees complete the CIHR sex and gender module and access available workshops and seminars (eg, The Libin Cardiovascular Institute’s Research is Better with Sex and Gender Symposium, Canadian Women’s Heart Health Summit). Frequently, new updates to sex and gender training resources for trainees such as in the recently funded CIHR training programs (https://cihr-irsc.gc.ca/e/52854.html) and the CIHR chronic disease networks (https://cihr-irsc.gc.ca/e/45854.html) and these can be accessed by visiting the Web site of each training platform. Through the expansion and diversity of training offers, more trainees will have access to these courses and achieve up to date knowledge.

Time and funding constraints might limit the scope and size of student-led projects such that the projects cannot be adequately powered to probe for sex differences. However, PIs should encourage their trainees to consider the possible effect of sex on results, interpretations, and conclusions as the minimum requirements of the Sex And Gender Equity in Research (SAGER) guidelines. Furthermore, senior research trainees will benefit from cowriting the sex and gender consideration sections of operating grant applications with their mentors. An initiative that individual departments or faculty could undertake to reduce funding barriers and enhance uptake by supervisors and trainees would be to offer small internal grants for student-led projects focused on sex and gender considerations.

## Intersectionality and Research in Women

***CVD and their risk factors are increasing among Indigenous and racialized women, while research and literature are sorely limited.***

### Indigenous women’s experiences of CVD

While women’s specific experiences of cardiac health outcomes are becoming more evident, a comprehensive understanding of Indigenous women’s experience of CVD in Canada is sorely lacking. CVD and risk factors continue to increase among Indigenous women. Undermining a clear understanding of Indigenous women’s experiences is the fact that most research is limited to general estimates of CVD or risk factors (Table 2). The historic marginalization, research ethical breaches, colonial mindsets, and lack of cultural competence in health care delivery and research involving Indigenous peoples has limited the trust and engagement of Indigenous peoples in the health research enterprise.^31^

**Table 2:** Publications reporting on sex differences or sex-specific experiences of cardiac outcomes among Indigenous women and men in Canada: 2001-2022

	Citation	Methods	Main outcomes	Indigenous population
CVD (general or unspecified)	First Nations Regional Longitudinal Health Survey (RHS) 2002/03. First Nations Centre: Ottawa, Ontario, 2007. Available at: https://www.nccih.ca/634/First_Nations_Regional_Longitudinal_Health_Survey_(RHS)_2002_03__Results_for_adults,_youth_and_child....nccih?id=1487&col=5. Accessed February 12, 2024.	Self-reported heart disease • n = 11,043 adults	• Women = 8.0% • Men = 7.3%	First Nations living in First Nations communities
	Ayotte P, Carrier A, Ouellet N, et al. Relation between methylmercury exposure and plasma paraoxonase activity in Inuit adults from Nunavik. Environ Health Persp 2011;119:1077-83.	Self-reported CVD • n = 491 Women • n = 405 Men	• Women = 19.7% • Men = 16.6%	Inuit of Nunavik
	Bombak AE. Predictors of Self-Rated Health in a Manitoba First Nation Community. Ann Arbor: University of Manitoba (Canada), 2010. Available at: https://iportal.usask.ca/record/47997. Accessed February 12, 2024.	Self-reported heart problems • n = 90 Women • n = 85 Men	• Women = 7% • Men = 27%	Manitoba First Nations
	Dewailly E, Chateau-Degat ML, Ékoe JM, Ladouceu R. Institut national de santé publique Quebec. Status of Cardiovascular Disease and Diabetes in Nunavik, 2007. Available at: http://www.santecom.qc.ca/Bibliothequevirtuelle/INSPQ/9782550506393.pdf. Accessed February 12, 2024.	Self-reported “other heart disease, not stroke or myocardial infarction” • n = 925 Women and Men	• Women = 6.1% • Men = 7.2%	Inuit of Nunavik
	Erber E, Beck L, De Roose E, Sharma S. Prevalence and risk factors for self-reported chronic disease amongst Inuvialuit populations. J Hum Nutr Diet 2010;23(suppl 1):43-50.	Self-reported heart disease • n = 175 women • n = 53 men	• Women = 6% • Men = 4%	Inuvialuit Inuit
	First Nations Regional Health Survey (RHS) 2008/10. First Nations Information Governance Centre: Ottawa, Ontario, 2012. Available at: https://fnigc.ca/wp-content/uploads/2020/09/5eedd1ce8f5784a69126edda537dccfc_first_nations_regional_health_survey_rhs_2008-10_-_national_report_adult_2.pdf. Accessed February 12, 2024.	Self-reported heart disease • n = 11,043 adults	• Women = 4.2% • Men = 6.7%	First Nations living in First Nations communities
	Foulds HJ, Bredin SS, Warburton DE. An evaluation of the physical activity and health status of British Columbian Aboriginal populations. Appl Physiol Nutr Metab 2012;37:127-37.	Self-reported CVD • n = 744 Women • n = 247 Men	• Women = 4.0% • Men = 10.0%	First Nations and Métis in British Columbia
	Foulds HJA, Bredin SSD, Warburton DER. The vascular health status of a population of adult Canadian Indigenous peoples from British Columbia. J Hum Hypertens 2016;30:278-84.	Self-reported CVD • n = 29 Female • n = 26 Male	• Women = 0.0% • Men = 0.0%	First Nations and Métis in British Columbia
	Gomes Z, Hart D, Downey B. Indigenous women’s perspectives on heart health and well-being: a scoping review. CJC Open 2022;5:43-53.	Review including 10 articles	• Women	Aboriginal and/or Torres Strait Islander, American Indian, First Nations and/or Métis, and Indigenous
	Hu XF, Singh K, Kenny TA, Chan HM. Prevalence of heart attack and stroke and associated risk factors among Inuit in Canada: a comparison with the general Canadian population. Int J Hyg Environ Health 2019:222:319-26.	Self-reported hypertension • n = 1270 Female Inuit • n = 1832 Female general Canadian • n = 800 Male Inuit • n = 1622 Male general Canadian	20-39 years • Female Inuit = 7.5% • Female general Canadian = 2.5% • Male Inuit = 12.2% • Male general Canadian = 2.5% 40-59 years • Female Inuit = 26.1% • Female general Canadian = 19.3% • Male Inuit = 24.0% • Male general Canadian = 21.3% 60-79 years • Female Inuit = 52.5% • Female general Canadian = 56.7% • Male Inuit = 54.8% • Male general Canadian = 58.0% Age-standardized • Female Inuit = 25.1% • Female general Canadian = 20.9% • Male Inuit = 24.8% • Male general Canadian = 21.4%	Canadian Inuit Nunangat, Nunatsiavut, Nunavut, and Inuvialuit Settlement region
	King KM, Sanguins J, McGregor L, LeBlanc P. First Nations people’s challenge in managing coronary artery disease risk. Qual Health Res 2007;17:1074-87.	Qualitative interviews • n = 11 women • n = 11 men	“Caregivers take care of everyone but themselves. Women are caregivers” Body image a powerful motivator for weight management for women	First Nations from Alberta, Saskatchewan, and Manitoba
	Medved MI, Brockmeier J, Morach J, Chartier-Courchene L. Broken heart stories: understanding Aboriginal women’s cardiac problems. Qual Health Res 2013;23:1613-25.	Qualitative interviews • n = 2 Women	CVD derived from “community imbalance”	Anishinaabe First Nations, Cree First Nations
	Park J, Tjepkema M, Goedhuis N, Pennock J. Avoidable mortality among First Nations adults in Canada: a cohort analysis. Health Rep 2015;26:10-6.	Avoidable death from diseases of circulatory system from Mortality Database • n = 34,420 women • n = 26,800 men	• Women = 22.3% • Men = 28.8%	Self-declared Indigenous in Canada
	Pollex RL, Hanley AJ, Zinman B, Harris SB, Khan HM, Hegele RA. Metabolic syndrome in aboriginal Canadians: prevalence and genetic associations. Atherosclerosis 2006;184:P121-9.	Hypertension • n = 295 female adults • n = 220 male adults • n = 115 female adolescents • n = 70 male adolescents	• Female adults = 9.2% • Male adults = 11.4% • Female adolescents = 0.87% • Male adolescents = 0.0%	Sandy Lake Oji-Cree First Nation
	Tjepkema M, Wilkins R, Goedhuis N, Pennock J. Cardiovascular disease mortality among First Nations people in Canada, 1991-2001. Chronic Dis Inj Can 2012;32:200-7.	CVD, “other CVD,” inflammatory heart disease, hypertensive heart disease mortality • n = 2317 Women • n = 2633 Men	Age Standardized Mortality Rate • CVD ▫ Women = 164.9 per 100,000 person-years ▫ Men = 250.2 per 100,000 person-years • “Other” CVD ▫ Women = 49.8 per 100,000 person-years ▫ Men = 58.1 per 100,000 person-years • Inflammatory heart disease ▫ Women = 3.5 per 100,000 person-years ▫ Men = 7.6 per 100,000 person-years • Hypertensive heart disease ▫ Women = 4.0 per 100,000 person-years ▫ Men = 2.5 per 100,000 person-years	Self-declared Indigenous in Canada
Pregnancy	Liu S, Zuberi SA, Malik AA, et al. Peripartum cardiomyopathy characteristics and outcomes in Canadian aboriginal and non-Aboriginal women. Can J Cardiol 2017;33:471-7.	Transthoracic echocardiography assessments of LVEF • n = 12 women with peripartum cardiomyopathy	• LVEF = 20% (IQR, 15%-23%) • LVEDD = 64 (IQR, 57-74) mm • LVESD = 56 (IQR, 48-67) mm	Status First Nations from Manitoba, Nunavut, and Ontario
	McGrath CK, Pudwell J, Pienaar E, Pienaar M, Smith GN. Cardiovascular risk screening and pregnancy complications: a comparison of two Canadian maternal health clinic populations. J Obstet Gynaecol Can 2021;43:1395-405.	Hospital/clinic records • n = 54 pregnant women	Hypertensive disorder (preeclampsia, HELLP syndrome, gestational hypertension • 42.6% hypertensive disorder	Status First Nations
Arrhythmias	Atzema CL, Kapral M, Klein-Geltink J, Asllani E. Cardiovascular disease in the Métis Nation of Ontario. Technical Report, 2012. Available at: https://www.metisnation.org/wp-content/uploads/2020/04/81920final20cvd20technical20en.pdf. Accessed February 12, 2024.	Discharge records • n = 5823 women • n = 6727 men	Atrial fibrillation • Women = 0.2% • Men = 0.4%	Métis in Ontario
	Huisman LA, Bene Watts S, Arbour L, McCormick R. Understanding the personal and community impact of long QT syndrome: a perspective from Gitxsan women. J Genet Couns 2020;29:562-73.	Qualitative interviews, photovoice, talking circles • n = 10 Women	• LQTS diagnosis traumatic • Positive family relationships, spirituality, knowledge about LQTS facilitate resiliency and coping • Poor understanding, conflicting medical advice and LQTS not being taken seriously by social contacts and healthcare providers hinder resiliency and coping • Learning to live with LQTS ongoing process; requires balance and interconnectedness between all aspects of well-being	Gitxsan First Nations
Atherosclerosis	Anand SS, Yusuf S, Jacobs R, et al. Risk factors, atherosclerosis, and cardiovascular disease among Aboriginal people in Canada: the Study of Health Assessment and Risk Evaluation in Aboriginal Peoples (Share-AP). Lancet 2001;358:1147-53.	Extracranial carotid artery atherosclerosis ultrasound IMT • n = 178 Female • n = 123 Male	Female participants less likely to have atherosclerosis • β Coefficient = −0.07 (standard error, 0.02)	Six Nations First Nations
	Dewailly E, Chateau-Degat ML, Ékoe JM, Ladouceu R. Institut national de santé publique Quebec. Status of Cardiovascular Disease and Diabetes in Nunavik, 2007. Available at: http://www.santecom.qc.ca/Bibliothequevirtuelle/INSPQ/9782550506393.pdf. Accessed February 12, 2024.	Carotid ultrasound IMT • n = 925 women and men	• Women ▫ 40-44 years = 0.53 ± 0.01 mm ▫ 45-54 years = 0.59 ± 0.01 mm ▫ 55-74 years = 0.81 ± 0.03 mm • Men ▫ 40-44 years = 0.59 ± 0.02 mm ▫ 45-54 years = 0.67 ± 0.03 mm ▫ 55-74 years = 0.93 ± 0.03 mm	Inuit of Nunavik
	Foulds HJ, Bredin SS, Warburton DE. The vascular health status of a population of adult Canadian Indigenous peoples from British Columbia. J Hum Hypertens 2016;30:278-84.	Carotid ultrasound IMT Central PWV Small and large arterial compliance • n = 29 Female • n = 26 Male	• Female ▫ IMT = 0.57 ± 0.08 mm ▫ PWV = 5.1 ± 2.3 m.s^-2^ ▫ Small arterial compliance = 6.4 ± 2.3 mL/mm Hg − 1 × 100 ▫ Large arterial compliance = 15.0 ± 5.9 mL/mm Hg − 1 × 10 • Male ▫ IMT = 0.61 ± 0.14 mm PWV = 5.7 ± 2.5 m۰s^-2^ ▫ Small arterial compliance = 8.9 ± 3.7 mL/mm Hg − 1 × 100 ▫ Large arterial compliance = 19.1 ± 9.8 mL/mm Hg − 1 × 10	First Nations and Métis in British Columbia
Myocardial infarction	Dewailly E, Chateau-Degat ML, Ékoe JM, Ladouceu R. Institut national de santé publique Quebec. Status of Cardiovascular Disease and Diabetes in Nunavik, 2007. Available at: http://www.santecom.qc.ca/Bibliothequevirtuelle/INSPQ/9782550506393.pdf. Accessed February 12, 2024.	Self-reported • n = 925 women and men	• Women = 2.1% • Men = 2.5%	Inuit of Nunavik
	Hu XF, Singh K, Kenny TA, Chan HM. Prevalence of heart attack and stroke and associated risk factors among Inuit in Canada: a comparison with the general Canadian population. Int J Hyg Environ Health 2019:222:319-26.	Self-reported • n = 1270 Female Inuit • n = 1832 Female general Canadian • n = 800 Male Inuit • n = 1622 Male general Canadian	20-39 Years • Female Inuit = 0.6% • Male Inuit = 0.5% • Male general Canadian = 0.0% 40-59 Years • Female Inuit = 2.6% • Female general Canadian = 1.8% • Male Inuit = 4.7% • Male general Canadian = 3.9% 60-79 Years • Female Inuit = 6.6% • Female general Canadian = 4.6% • Male Inuit = 14.6% • Male general Canadian = 11.3% Age-standardized • Female Inuit = 3.1% • Female general Canadian = 1.8% • Male Inuit = 5.0% • Male general Canadian = 3.9%	Canadian Inuit Nunangat, Nunatsiavut, Nunavut, and Inuvialuit Settlement region

	Tjepkema M, Wilkins R, Goedhuis N, Pennock J. Cardiovascular disease mortality among First Nations people in Canada, 1991-2001. Chronic Dis Inj Can 2012;32:200-7.	Acute myocardial infarction mortality • n = 2317 women • n = 2633 men	Age-standardized mortality rate • Women = 38.8 per 100,000 person-years • Men = 82.2 per 100,000 person-years	Self-declared Indigenous in Canada

Stroke	Atzema CL, Kapral M, Klein-Geltink J, Asllani E. Cardiovascular disease in the Métis Nation of Ontario. Technical Report, 2012. Available at: https://www.metisnation.org/wp-content/uploads/2020/04/81920final20cvd20technical20en.pdf. Accessed February 12, 2024.	Discharge Records • n = 5823 women • n = 6727 men	• Women = 0.2% • Men = 0.4%	Métis in Ontario
	Dewailly E, Chateau-Degat ML, Ékoe JM, Ladouceu R. Institut national de santé publique Quebec. Status of Cardiovascular Disease and Diabetes in Nunavik, 2007. Available at: http://www.santecom.qc.ca/Bibliothequevirtuelle/INSPQ/9782550506393.pdf. Accessed February 12, 2024.	Self-reported • n = 925 women and men	• Women = 3.6% • Men = 4.6%	Inuit of Nunavik
	Hu XF, Singh K, Kenny TA, Chan HM. Prevalence of heart attack and stroke and associated risk factors among Inuit in Canada: a comparison with the general Canadian population. Int J Hyg Environ Health 2019:222:319-26.	Self-reported • n = 1270 Female Inuit • n = 1832 Female general Canadian • n = 800 Male Inuit • n = 1622 Male general Canadian	20-39 Years • Female Inuit = 1.1% • Female general Canadian = 0.0% • Male Inuit = 1.2% 40-59 Years • Female Inuit = 2.5% • Male Inuit = 2.9% 60-79 Years • Female Inuit = 3.5% • Female general Canadian = 2.9% • Male Inuit = 3.2% • Male general Canadian = 2.8% Age-standardized • Female Inuit = 2.2% • Female general Canadian = 1.0% • Male Inuit = 2.1% • Male general Canadian = 0.8%	Canadian Inuit Nunangat, Nunatsiavut, Nunavut, and Inuvialuit Settlement region
	Tjepkema M, Wilkins R, Goedhuis N, Pennock J. Cardiovascular disease mortality among First Nations people in Canada, 1991-2001. Chronic Dis Inj Can 2012;32:200-7.	Cerebrovascular disease and specifically stroke mortality • n = 2317 Women • n = 2633 Men	Age-standardized mortality rate • Cerebrovascular disease ▫ Women = 41.7 per 100,000 person-years ▫ Men = 35.3 per 100,000 person-years • Stroke ▫ Women = 37.0 per 100,000 person-years ▫ Men = 30.7 per 100,000 person-years	Self-declared Indigenous in Canada

Coronary artery diseases	Atzema CL, Kapral M, Klein-Geltink J, Asllani E. Cardiovascular disease in the Métis Nation of Ontario. Technical Report, 2012. Available at: https://www.metisnation.org/wp-content/uploads/2020/04/81920final20cvd20technical20en.pdf. Accessed February 12, 2024.	Discharge records • n = 5823 women • n = 6727 men	Acute coronary syndromes • Women = 1% • Men = 1.8%	Métis in Ontario

Heart failure	Atzema CL, Kapral M, Klein-Geltink J, Asllani E. Cardiovascular disease in the Métis Nation of Ontario. Technical Report, 2012. Available at: https://www.metisnation.org/wp-content/uploads/2020/04/81920final20cvd20technical20en.pdf. Accessed February 12, 2024.	Discharge records • n = 5823 women • n = 6727 men	Congestive heart failure • Women = 0.8% • Men = 1.3%	Métis in Ontario
	Lyons KJ, Ezekowitz JA, Liu W, McAlister FA, Kaul P. Mortality outcomes among status Aboriginals and whites with heart failure. Can J Cardiol 2014;30:619-26.	1-Year mortality among patients with incident heart failure • n = 583 women • n = 575 men	Odds ratios for men • 1.11 (95% CI, 1.06-1.16)	First Nations from Alberta

Congenital heart disease	Atzema CL, Kapral M, Klein-Geltink J, Asllani E. Cardiovascular disease in the Métis Nation of Ontario. Technical Report, 2012. Available at: https://www.metisnation.org/wp-content/uploads/2020/04/81920final20cvd20technical20en.pdf. Accessed February 12, 2024.	Discharge records • n = 5823 women • n = 6727 men	• Women = 0% • Men = 0%	Métis in Ontario
	Tjepkema M, Wilkins R, Goedhuis N, Pennock J. Cardiovascular disease mortality among First Nations people in Canada, 1991-2001. Chronic Dis Inj Can 2012;32:200-7.	Congestive heart failure mortality • n = 2317 women • n = 2633 men	Age-standardized mortality rate • Women = 10.3 per 100,000 person-years • Men = 10.8 per 100,000 person-years	Self-declared Indigenous in Canada
Ischemic heart disease	Tjepkema M, Wilkins R, Goedhuis N, Pennock J. Cardiovascular disease mortality among First Nations people in Canada, 1991-2001. Chronic Dis Inj Can 2012;32:200-7.	Ischemic heart disease mortality • n = 2317 Women • n = 2633 Men	Age-standardized mortality rate • Women = 44.6 per 100,000 person-years • Men = 62.4 per 100,000 person-years	Self-declared Indigenous in Canada
Rheumatic heart disease	Atzema CL, Kapral M, Klein-Geltink J, Asllani E. Cardiovascular disease in the Métis Nation of Ontario. Technical Report, 2012. Available at: https://www.metisnation.org/wp-content/uploads/2020/04/81920final20cvd20technical20en.pdf. Accessed February 12, 2024.	Discharge records • n = 5823 women • n = 6727 men	• Women = 0% • Men = 0%	Métis in Ontario
	Tjepkema M, Wilkins R, Goedhuis N, Pennock J. Cardiovascular disease mortality among First Nations people in Canada, 1991-2001. Chronic Dis Inj Can 2012;32:200-7.	Rheumatic heart disease mortality • n = 2317 Women • n = 2633 Men	Age-standardized mortality rate • Women = 3.2 per 100,000 person-years • Men = 3.1 per 100,000 person-years	Self-declared Indigenous in Canada

CI, confidence interval; CVD, cardiovascular disease; HELLP, hemolysis, elevated liver enzymes and low platelets; IMT, intima-media thickness; LQTS, long QT syndrome; LLVEDD, left ventricular end diastolic diameter; LVEF, left ventricular ejection fraction; LVESD, left ventricular end systolic diameter; PWV, pulse wave velocity.

The limited literature around Indigenous women’s experiences of CVD represent incomplete assessments across Indigenous identities. Although broad terms such as Indigenous and Aboriginal are often used, these terms obscure significant diversity across hundreds of Indigenous communities representing more than 60 distinct Indigenous languages (https://www12.statcan.gc.ca/census-recensement/2021/dp-pd/dv-vd/language-langue/index-en.html). Considerable diversity exists across Indigenous communities, including diversity of health outcomes and CVD risk factors.^32^ Approximately 28% of this diversity in risk factors and health outcomes can be attributed to socioeconomic status, access to health care, access to affordable prescription medications, educational opportunities, social supports, and community cohesion.^33^ Further compounding the lack of data available is the inconsistent accuracy and reporting of identity. Among available literature, some articles incorrectly or incompletely identify participant identity, use flawed methods of identifying Indigenous participants through census self-identification, or use a pan-Indigenous approach of combining multiple broad identities without reporting results among specific groups (Table 2).^34^, ^35^, ^36^, ^37^, ^38^, ^39^ Addressing gaps in the literature of Indigenous women’s experiences of CVD will require greater understanding of experiences of Indigenous women from diverse Indigenous nations with clear and accurate descriptions of identity, and requires individual reporting of data according to identity or nation. Moreover, improving environmental conditions would be of major importance to improve health in remote communities.^40^

### Racialized women’s experiences of CVD

The 2021 Statistics Canada census data on race and ethnicity indicates that the total racialized peoples population in Canada comprises 26.5% of the overall population, the largest group being of South Asian origin, followed by Chinese and Black populations. Each racialized group includes approximately 50% women (https://www12.statcan.gc.ca/census-recensement/2021/dp-pd/prof/index.cfm?Lang%20=%20E). The confluence of identifying as racialized and as a woman represents a particularly under-represented group in CVD research. Within Canada, comprehensive understandings of the experiences of CVD among racialized communities, including African descent, East Asian, and South Asian individuals are sparse. Amidst the limited literature in this area, very few report results specific to the experiences of women.

Across different ethnic identities, racialized women’s experiences of CVD vary widely (Table 3). Generally, East Asian women experience lower rates of CVD, whereas South Asian women and women of African descent experience higher rates of CVD.^41^, ^42^, ^43^ Conversely, women of Filipina descent experience greater risks of hypertension than East Asian women and women of European descent.^44^ The experiences of women of African descent are much more varied, particularly in comparison with women of European descent, with women of African descent having lower rates of CAD^42^ and stroke,^41^ and higher rates of hypertension.^41^^,^^43^^,^^45^ The sparse literature of South Asian women’s specific experiences identifies a higher rate of CAD mortality and incidence of acute myocardial infarction, compared with women of European descent in Canada.^46^ Immigrating to Canada affects racialized women’s experiences of CVD, with increases in experiences of hypertension with immigration, when women of African descent, and South Asian women experience the greatest increases in hypertension, whereas women of European descent and Chinese women experience minimal changes in hypertension prevalence with immigration to Canada.^41^

**Table 3:** Publications reporting sex-differences or sex-specific experiences of cardiac outcomes among nonwhite women and men in Canada: 2001-2022

Cardiovascular health	Citation	Methods	Main outcomes	Racialized community
CVD (general or unspecified)	Chen G, Khan N, King KM, Hemmelgarn BR, Quan H. Home care utilization and outcomes among Asian and other Canadian patients with heart failure. BMC Cardiovasc Disord 2010;10:12.	Home care service records • n = 635 Asian female • n = 547 Asian male • n = 11,504 Other Canadian Female • n = 13,485 Other Canadian Male	Home care service utilization by individuals with heart failure • Asian female = 66.0% • Asian male = 44.8% • Other Canadian Female = 70.4% • Other Canadian Male = 48.8%	Asian

	Chiu M, Austin PC, Manuel DG, Tu JV. Comparison of cardiovascular risk profiles among ethnic groups using population health surveys between 1996 and 2007. CMAJ 2010;182:E301-10.	Self-reported hypertension, heart disease, and stroke • n = 1570 Chinese women • n = 1623 South Asian women • n = 1528 women of African descent • n = 84,098 women of European descent • n = 1468 Chinese men • n = 1741 South Asian men • n = 1214 men of African descent • n = 74,978 men of European descent	Hypertension • Chinese women = 15.8% • South Asian women = 17.9% • Women of African descent = 21.7% • Women of European descent = 14.8% • Chinese men = 14.4% • South Asian men = 16.1% • Men of African descent = 17.7% • Men of European descent = 13.1% Heart disease • Chinese women = 2.7% • South Asian women = 5.2% • Women of African descent = 4.2% • Women of European descent = 4.7% • Chinese men = 3.8% • South Asian men = 5.2% • Men of African descent = 2.5% • Men of European descent = 5.4% Heart disease or stroke • Chinese women = 3.5% • South Asian women = 7.1% • Women of African descent = 4.9% • Women of European descent = 5.4% • Chinese men = 4.1% • South Asian men = 6.0% • Men of African descent = 3.8% • Men of European descent = 6.0%	African descent, Chinese, South Asian

	Chiu M, Austin PC, Manuel DG, Tu JV. Cardiovascular risk factor profiles of recent immigrants vs long-term residents of Ontario: a multi-ethnic study. Can J Cardiol 2012;28:20-6.	National Population Health Survey (1996) and Canadian Community Health Surveys (2001, 2003, 2005, 2007) • n = 521 African descent women recent immigrants • n = 1007 African descent women long-term resident • n = 772 Chinese women recent immigrants • n = 798 Chinese women long-term residents • n = 887 South Asian women recent immigrants • n = 736 South Asian women long-term residents • n = 1819 European descent women recent immigrants • n = 82,279 European descent women long-term residents • n = 379 African descent men recent immigrants • n = 835 African descent men long-term residents • n = 703 Chinese men recent immigrants • n = 765 Chinese men long-term residents • n = 904 South Asian men recent immigrants • n = 837 South Asian men long-term residents • n = 1530 European descent men recent immigrants • n = 69,025 European descent men long-term residents	Self-reported Hypertension • African descent women recent immigrants = 13.9% • African descent women long-term residents = 24.3% • Chinese women recent immigrants = 16.4% • Chinese women long-term residents = 16.1% • South Asian women recent immigrants = 14.7% • South Asian women long-term residents = 18.1% • European descent women recent immigrants = 14.5% • European descent women long-term residents = 14.4% • African descent men recent immigrants = 16.1% • African descent men long-term residents = 17.7% • Chinese men recent immigrants = 12.5% • Chinese men long-term residents = 15.3% • South Asian men recent immigrants = 14.7% • South Asian men long-term residents = 15.3% • European descent men recent immigrants = 11.9% • European descent men long-term residents = 12.9%	African descent, Chinese, South Asian
	Di Giuseppe G, Chu A, Tu JV, Shanmugasegaram S, Liu P, Lee DS. Incidence of heart failure among immigrants to Ontario, Canada: a CANHEART immigrant study. J Card Fail 2019;25:425-35.	Administrative data • n = 97,078 East Asian women • n = 47,679 Southeast Asian women • n = 29,875 Latin American women • n = 38,396 Western European women • n = 42,484 African descent women • n = 94,286 South Asian women • n = 36,771 West Asian/Arab women • n = 89,893 East Asian men • n = 30,161 Southeast Asian men • n = 28,974 Latin American men • n = 61,361 Eastern European men • n = 39,225 Western European men • n = 40,623 African descent men • n = 106,880 South Asian men • n = 46,938 West Asian/Arab men	Hypertension (%) • East Asian women = 28.9 (28.5-29.3) • Southeast Asian women = 42.8 (42.1-43.5) • Latin American women = 35.7 (34.8-36.5) • Eastern European women = 32.8 (32.3-33.4) • Western European women = 30.4 (29.7-31.1) • African descent women = 46.9 (46.1-47.7) • South Asian women = 36.8 (36.4-37.3) • West Asian/Arab women = 30.4 (29.7-31.1) • East Asian men = 25.2 (24.8-25.5) • Southeast Asian men = 42.1 (41.2-42.9) • Latin American men = 30.7 (29.8-31.5) • Eastern European men = 30.5 (30.0-31.1) • Western European men = 27.5 (26.9-28.2) • African descent men = 37.4 (36.6-38.3) • South Asian men = 35.5 (35.1-35.9) • West Asian/Arab men = 26.6 (26.0-27.2)	East Asian, Southeast Asian, Latin American, Eastern European, Western European, African descent, South Asian, West Asian/Arab

	Foulds HJ, Bredin SS, Warburton DE. Greater prevalence of select chronic conditions among Aboriginal and South Asian participants from an ethnically diverse convenience sample of British Columbians. Appl Physiol Nutr Metab 2012;37:1212-21.	Self-report hypertension, CVD, measured blood pressure • n = 112 South Asian women • n = 279 East Asian women • n = 2187 women of European descent • n = 116 South Asian men • n = 187 East Asian men • n = 1463 men of European descent	Hypertension • South Asian women = 16.1% • East Asian women = 3.2% • Women of European descent = 22.6% • South Asian men = 27.6% • East Asian men = 23.7% • Men of European descent = 28.0% CVD • South Asian women = 1.8% • East Asian women = 0.7% • Women of European descent = 3.6% • South Asian men = 6.9% • East Asian men = 1.1% • Men of European descent = 8.7%	South Asian, East Asian
	Fuller-Thomson E, Rotermann M, Ray JG. Elevated risk factors for adverse pregnancy outcomes among Filipina-Canadian women. J Obstet Gynaecol Can 2010;32:113-9.	Canadian Community Health Survey Self-reported chronic hypertension • n = 115,842 Filipina women • n = 394,357 East Asian women • n = 5,812,851 women of European descent	Hypertension • Filipina women = 7.1% • East Asian women = 3.9% • Women of European descent = 4.8%	

	King-Shier KM, Singh S, LeBlanc P, et al. The influence of ethnicity and gender on navigating an acute coronary syndrome event. Eur J Cardiovasc Nurs 2015;14:240-7.	Qualitative interviews • n = 8 Chinese women • n = 9 South Asian women • n = 10 women of European descent • n = 10 Chinese men • n = 10 South Asian men • n = 10 men of European descent	Unstable angina • Chinese women = 62.5% • South Asian women = 50.0% • Women of European descent = 10.0% • Chinese men = 20.0% • South Asian men = 40.0% • Men of European descent = 60.0% Chinese and South Asian people more likely to delay care-seeking Chinese and South Asian women’s families reluctant to acknowledge woman’s need to seek health care, take her to obtain health care, or allow her to be admitted to hospital to receive health care	Chinese, South Asian

	Miao Q, Dunn S, Wen SW, et al. Association of maternal socioeconomic status and race with risk of congenital heart disease: a population-based retrospective cohort study in Ontario, Canada. BMJ Open 2022;12:e051020.	Discharge Abstract Database and National Ambulatory Care Reporting System: chronic hypertension and heart disease • n = 35,258 women of African descent • n = 139,895 Asian women • n = 315,919 women of European descent	Chronic hypertension • Asian women = 0.88% • Women of African descent = 2.08% • Women of European descent = 0.93% Heart disease • Asian women = 1.33% • Women of African descent = 2.89% • Women of European descent = 2.36% Likelihood of developing CHD (Adjusted HR 95%CI) • Asian women = 0.83 (0.78-0.88) • Women of African descent = 1.36 (1.24-1.50)	African descent, Asian

	Quan H, Chen G, Walker RL, et al. Incidence, cardiovascular complications and mortality of hypertension by sex and ethnicity. Heart 2013;99:715-21.	Administrative data of patients with newly diagnosed hypertension • n = 25,914 Chinese women • n = 23,978 Chinese men • n = 19,833 South Asian women • n = 19,342 South Asian men • n = 438,021 women of European descent • n = 403,256 men of European descent	All-cause mortality • Chinese women = 12.2 (11.7-12.8) per 100 person years • South Asian women = 18.0 (17.2-18.8) per 100 person years • European descent women = 21.8 (21.6-22.0) per 100 person years • Chinese men = 12.8 (12.2-13.4) per 100 person years • South Asian men = 20.0 (19.1-20.9) per 100 person years • European descent men = 26.5 (26.2-26.7) per 100 person years CVD • Chinese women = 8.7 (8.2-9.2) per 100 person years • South Asian women = 15.9 (15.1-16.7) per 100 person years • European descent women = 18.5 (18.4-18.7) per 100 person years • Chinese men = 10.5 (9.9-11.1) per 100 person years • South Asian men = 21.0 (20.1-22.0) per 100 person years • European descent men = 24.7 (24.5-25.0) per 100 person years	Chinese, South Asian
	Subhan FB, Chan CB. Diet quality and risk factors for cardiovascular disease among South Asians in Alberta. Appl Physiol Nutr Metab 2019;44:886-93.	Self-reported hypertension • n = 72 women • n = 68 men	Self-reported hypertension • Women = 12.5% • Men = 15%	South Asian

	Veenstra G, Patterson AC. Black-white health inequalities in Canada. J Immigr Minor Health 2016;18:51-7.	Self-reported heart disease and hypertension • n = 3127 women of African descent • n = 309,720 women of European descent • n = 2529 men of African descent • n = 250,511 men of European descent	Heart disease • Women of African descent = 3% • Women of European descent = 7% • Men of African descent = 3% • Men of European descent = 9% Hypertension • Women of African descent = 23% • Women of European descent = 26% • Men of African descent = 19% • Men of European descent = 22% Odds ratios of ill health with hypertension • Women of African descent = 2.52 (2.07-3.06) Odds ratios of ill health with heart disease • Women of African descent = 0.68 (0.46-1.02)	African descent

Atrial fibrillation	Di Giuseppe G, Chu A, Tu JV, Shanmugasegaram S, Liu P, Lee DS. Incidence of heart failure among immigrants to Ontario, Canada: a CANHEART immigrant study. J Card Fail 2019;25:425-35.	Administrative data • n = 97,078 East Asian women • n = 47,679 Southeast Asian women • n = 29,875 Latin American women • n = 65,199 Eastern European women • n = 38,396 Western European women • n = 42,484 African descent women • n = 94,286 South Asian women • n = 36,771 West Asian/Arab women • n = 89,893 East Asian men • n = 30,161 Southeast Asian men • n = 28,974 Latin American men • n = 61,361 Eastern European men • n = 39,225 Western European men • n = 40,623 African descent men • n = 106,880 South Asian men • n = 46,938 West Asian/Arab men	• East Asian women = 0.9 (0.9-1.0)% • Southeast Asian women = 0.9 (0.8-1.0)% • Latin American women = 0.9 (0.8-1.1)% • Eastern European women = 2.0 (1.9-2.2)% • Western European women = 1.6 (1.5-1.8)% • African descent women = 0.9 (0.8-1.0)% • South Asian women = 0.7 (0.6-0.7)% • West Asian/Arab women = 1.3 (1.2-1.5)% • East Asian men = 0.9 (0.9-1.0)% • Southeast Asian men = 1.4 (1.2-1.6)% • Latin American men = 0.9 (0.8-1.1)% • Eastern European men = 2.3 (2.1-2.5)% • Western European men = 2.0 (1.8-2.2)% • African descent men = 0.8 (0.6-0.9)% • South Asian men = 0.7 (0.7-0.8)% • West Asian/Arab men = 1.4 (1.3-1.6)%	East Asian, Southeast Asian, Latin American, Eastern European, Western European, African descent, South Asian, West Asian/Arab
Myocardial infarction	Di Giuseppe G, Chu A, Tu JV, Shanmugasegaram S, Liu P, Lee DS. Incidence of heart failure among immigrants to Ontario, Canada: a CANHEART immigrant study. J Card Fail 2019;25:425-35.	Administrative data • n = 97,078 East Asian women • n = 47,679 Southeast Asian women • n = 29,875 Latin American women • n = 65,199 Eastern European women • n = 38,396 Western European women • n = 42,484 African descent women • n = 94,286 South Asian women • n = 36,771 West Asian/Arab women • n = 89,893 East Asian men • n = 30,161 Southeast Asian men • n = 28,974 Latin American men • n = 61,361 Eastern European men • n = 39,225 Western European men • n = 40,623 African descent men • n = 106,880 South Asian men • n = 46,938 West Asian/Arab men	• East Asian women = 0.2 (0.2-0.3)% • Southeast Asian women = 0.5 (0.4-0.6)% • Latin American women = 1.0 (0.8-1.1)% • Eastern European women = 0.7 (0.7-0.8)% • Western European women = 0.7 (0.6-0.8)% • African descent women = 0.7 (0.6-0.8) • South Asian women = 1.0 (1.0-1.1)% • West Asian/Arab women = 0.7 (0.6-0.9)% • East Asian men = 0.6 (0.5-0.6)% • Southeast Asian men = 1.5 (1.3-1.7)% • Latin American men = 2.3 (2.1-2.6)% • Eastern European men = 2.6 (2.4-2.7)% • Western European men = 2.0 (1.8-2.2)% • African descent men = 1.4 (1.3-1.6)% • South Asian men = 2.9 (2.8-3.1)% • West Asian/Arab men = 2.5 (2.3-2.7)%	East Asian, Southeast Asian, Latin American, Eastern European, Western European, African descent, South Asian, West Asian/Arab

	King-Shier KM, Singh S, LeBlanc P, et al. The influence of ethnicity and gender on navigating an acute coronary syndrome event. Eur J Cardiovasc Nurs 2015;14:240-7.	Qualitative interviews • n = 8 Chinese women • n = 9 South Asian women • n = 10 women of European descent • n = 10 Chinese men • n = 10 South Asian men • n = 10 men of European descent	• Chinese Women = 37.5% • South Asian Women = 40.0% • Women of European descent = 90.0% • Chinese men = 80.0% • South Asian men = 60.0% • Men of European descent = 40.0%	Chinese, South Asian

	Nijjar AP, Wang H, Quan H, Khan NA. Ethnic and sex differences in the incidence of hospitalized acute myocardial infarction: British Columbia, Canada 1995-2002. BMC Cardiovasc Disord 2010;10:38.	Administrative data • n = 102,805 Chinese women • n = 89,350 Chinese men • n = 44,175 South Asian women • n = 43,790 South Asian men • n = 978,553 women of European descent • n = 909,870 men of European descent	• Chinese Women = 0.49/1000 per year • South Asian Women = 2.35/1000 per year • Women of European descent = 1.53/1000 per year • Chinese men = 0.98/1000 per year • South Asian men = 4.97/1000 per year • Men of European descent = 3.29/1000 per year	Chinese, South Asian

	Quan H, Chen G, Walker RL, et al. Incidence, cardiovascular complications and mortality of hypertension by sex and ethnicity. Heart 2013;99:715-21.	Administrative data of patients with newly diagnosed hypertension • n = 25,914 Chinese women • n = 23,978 Chinese men • n = 19,833 South Asian women • n = 19,342 South Asian men • n = 438,021 women of European descent • n = 403,256 men of European descent	• Chinese women = 2.3 (2.0-2.5) per 100 person-years • South Asian women = 5.6 (5.1-6.0) per 100 person-years • European descent women = 6.4 (6.3-6.5) per 100 person-years • Chinese men = 4.3 (3.9-4.7) per 100 person-years • South Asian men = 9.7 (9.1-10.4) per 100 person-years • European descent men = 11.3 (11.2-11.5) per 100 person-years	Chinese, South Asian

Stroke	Chiu M, Austin PC, Manuel DG, Tu JV. Comparison of cardiovascular risk profiles among ethnic groups using population health surveys between 1996 and 2007. CMAJ 2010;182:E301-10.	Self-reported stroke • n = 1570 Chinese women • n = 1623 South Asian women • n = 1528 Women of African descent • n = 84,098 women of European descent • n = 1468 Chinese men • n = 1741 South Asian men • n = 1214 men of African descent • n = 74,978 men of European descent	Stroke • Chinese women = 0.8% • South Asian women = 2.2% • Women of African descent = 1.1% • Women of European descent = 1.2% • Chinese men = 0.5% • South Asian men = 1.1% • Men of African descent = 1.4% • Men of European descent = 1.1%	African descent, Chinese, South Asian
	Quan H, Chen G, Walker RL, et al. Incidence, cardiovascular complications and mortality of hypertension by sex and ethnicity. Heart 2013;99:715-21.	Administrative data of patients with newly diagnosed hypertension • n = 25,914 Chinese women • n = 23,978 Chinese men • n = 19,833 South Asian women • n = 19,342 South Asian men • n = 438,021 women of European descent • n = 403,256 men of European descent	• Chinese women = 4.4 (4.1-4.8) per 100 person-years • South Asian women = 6.0 (5.5-6.5) per 100 person-years • European descent women = 7.2 (7.1-7.3) per 100 person-years • Chinese men = 4.6 (4.2-5.0) per 100 person-years • South Asian men = 6.7 (6.2-7.3) per 100 person-years • European descent men = 8.3 (8.1-8.4) per 100 person-years	Chinese, South Asian

Coronary artery diseases	Di Giuseppe G, Chu A, Tu JV, Shanmugasegaram S, Liu P, Lee DS. Incidence of heart failure among immigrants to Ontario, Canada: a CANHEART immigrant study. J Card Fail 2019;25:425-35.	Administrative data • n = 97,078 East Asian women • n = 47,679 Southeast Asian women • n = 29,875 Latin American women • n = 65,199 Eastern European women • n = 38,396 Western European women • n = 42,484 African descent women • n = 94,286 South Asian women • n = 36,771 West Asian/Arab women • n = 89,893 East Asian men • n = 30,161 Southeast Asian men • n = 28,974 Latin American men • n = 61,361 Eastern European men • n = 39,225 Western European men • n = 40,623 African descent men • n = 106,880 South Asian men • n = 46,938 West Asian/Arab men	Percutaneous coronary intervention or coronary artery bypass graft (%) • East Asian women = 0.2 (0.2-0.3) • Southeast Asian women = 0.5 (0.4-0.6) • Latin American women = 0.9 (0.8-1.0) • Eastern European women = 0.9 (0.8-1.0) • Western European women = 0.7 (0.6-0.8) • African descent women = 0.6 (0.5-0.7) • South Asian women = 1.2 (1.1-1.3) • West Asian/Arab women = 1.0 (0.9-1.2) • East Asian men = 0.8 (0.8-0.9) • Southeast Asian men = 2.0 (1.8-2.2) • Latin American men = 2.7 (2.4-3.0) • Eastern European men = 3.4 (3.2-3.6) • Western European men = 2.3 (2.1-2.5) • African descent men = 1.7 (1.5-1.9) • South Asian men = 4.4 (4.2-4.5) • West Asian/Arab men = 3.9 (3.7-4.2)	East Asian, Southeast Asian, Latin American, Eastern European, Western European, African descent, South Asian, West Asian/Arab

Heart failure	Di Giuseppe G, Chu A, Tu JV, Shanmugasegaram S, Liu P, Lee DS. Incidence of heart failure among immigrants to Ontario, Canada: a CANHEART immigrant study. J Card Fail 2019;25:425-35.	Administrative Data • n = 97,078 East Asian women • n = 47,679 Southeast Asian women • n = 29,875 Latin American women • n = 65,199 Eastern European women • n = 38,396 Western European women • n = 42,484 African descent women • n = 94,286 South Asian women • n = 36,771 West Asian/Arab women • n = 89,893 East Asian men • n = 30,161 Southeast Asian men • n = 28,974 Latin American men • n = 61,361 Eastern European men • n = 39,225 Western European men • n = 40,623 African descent men • n = 106,880 South Asian men • n = 46,938 West Asian/Arab men	Incidence, per 1000 person-years • East Asian women = 0.51 (0.44-0.60) • Southeast Asian women = 0.97 (0.81-1.15) • Latin American women = 1.16 (0.93-1.43) • Eastern European women = 1.34 (1.16-1.54) • Western European women = 1.41 (1.20-1.65) • African descent women = 1.26 (1.06-1.50) • South Asian women = 1.48 (1.33-1.63) • West Asian/Arab women = 1.60 (1.33-1.90) • East Asian men = 0.38 (0.32-0.45) • Southeast Asian men = 1.0 (0.80-1.23) • Latin American men = 0.94 (0.71-1.21) • Eastern European men = 1.06 (0.88-1.27) • Western European men = 0.98 (0.79-1.21) • African descent men = 1.19 (0.94-1.47) • South Asian men = 1.16 (1.05-1.29) • West Asian/Arab men = 1.14 (0.95-1.37)	East Asian, Southeast Asian, Latin American, Eastern European, Western European, African descent, South Asian, West Asian/Arab

	Quan H, Chen G, Walker RL, et al. Incidence, cardiovascular complications and mortality of hypertension by sex and ethnicity. Heart 2013;99:715-21.	Administrative data of patients with newly diagnosed hypertension • n = 25,914 Chinese women • n = 23,978 Chinese men • n = 19,833 South Asian women • n = 19,342 South Asian men • n = 438,021 women of European descent • n = 403,256 men of European descent	• Chinese women = 3.8 (3.5-4.1) per 100 person-years • South Asian women = 7.9 (7.3-8.5) per 100 person-years • European descent women = 9.0 (8.9-9.1) per 100 person-years • Chinese men = 4.0 (3.6-4.4) per 100 person-years • South Asian men = 8.2 (7.7-8.8) per 100 person-years • European descent men = 10.4 (10.3-10.6) per 100 person-years	Chinese, South Asian

Valvular heart disease	Di Giuseppe G, Chu A, Tu JV, Shanmugasegaram S, Liu P, Lee DS. Incidence of heart failure among immigrants to Ontario, Canada: a CANHEART immigrant study. J Card Fail 2019;25:425-35.	Administrative data • n = 97,078 East Asian women • n = 47,679 Southeast Asian women • n = 29,875 Latin American women • n = 65,199 Eastern European women • n = 38,396 Western European women • n = 42,484 African descent women • n = 94,286 South Asian women • n = 36,771 West Asian/Arab women • n = 89,893 East Asian men • n = 30,161 Southeast Asian men • n = 28,974 Latin American men • n = 61,361 Eastern European men • n = 39,225 Western European men • n = 40,623 African descent men • n = 106,880 South Asian men • n = 46,938 West Asian/Arab men	• East Asian women = 0.2 (0.2-0.3)% • Southeast Asian women = 0.3 (0.3-0.4)% • Latin American women = 0.4 (0.3-0.5)% • Eastern European women = 0.5 (0.4-0.5)% • Western European women = 0.5 (0.4-0.6)% • African descent women = 0.4 (0.3-0.5)% • South Asian women = 0.3 (0.3-0.4)% • West Asian/Arab women = 0.5 (0.4-0.6)% • East Asian men = 0.2 (0.1-0.2)% • Southeast Asian men = 0.3 (0.3-0.4)% • Latin American men = 0.3 (0.2-0.4)% • Eastern European men = 0.5 (0.4-0.6)% • Western European men = 0.5 (0.4-0.6)% • African descent men = 0.3 (0.2-0.4)% • South Asian men = 0.3 (0.3-0.4)% • West Asian/Arab men = 0.4 (0.3-0.5)%	East Asian, Southeast Asian, Latin American, Eastern European, Western European, African descent, South Asian, West Asian/Arab

CHD, chronic heart disease; CVD, cardiovascular disease.

Racialized women present different risk profiles and experiences of CVD compared with women of European descent in Canada. Understanding, managing, and treating CVD among racialized women requires understanding and evidence specific to their needs and experiences. Although a clear gap in literature of the CVD experiences among racialized women is evident, solutions require evidence specific to them, and reporting of women-specific data within studies including racialized men and women.

## Funding Agencies: Grants, Scholarships, and Awards

***Federal*** ***granting*** ***agencies now require integration of sex and gender as variables in all applications, or in lieu of this, presentation of a strong rationale as to why it is not required. Provincial and local*** ***granting*** ***agencies do not consistently have this requirement, and policy change to require the integration of sex and gender into all*** ***funding*** ***applications is recommended. Numerous*** ***funding*** ***opportunities exist to enhance the integration of sex and gender in research*** ***supported*** ***by federal*** ***granting*** ***agencies.***

In cellular mechanistic studies, it has been identified that sex specificity exists; indeed, “every cell has a sex” has been the mantra of the National Institute of Health since 2016, when a new policy was enacted requiring that preclinical research also consider sex as an important biological variable in vertebrate animal and human studies. This policy served as a complement to the inclusion policy requiring reporting analyses of sex, gender, racial, and ethnic differences in clinical trials established in 2001. These efforts have expanded the consideration of sex in research designs, analyses, and reporting across the research continuum, from preclinical studies to clinical randomized controlled trials.

The CIHR Institute of Gender and Health (IGH) has implemented multiple initiatives to increase sex- and gender-based analysis (SGBA; SGBA Plus [SGBA+]) in health research. These include IGH-specific initiatives and cross-cutting initiatives performed in collaboration with other government agencies. These programs are meant to promote the application of sex and gender outcomes in the current health research landscape (http://www.cihr-irsc.gc.ca/e/51310.html). Several societies and grant foundations also recognize the importance of integrating SGBA (HSFC, Canadian Cancer Society) and applying promotional strategies (www.heartandstroke.ca/women). In this section, we outline the current Canadian initiatives to fund and promote SGBA+ in health research.

### SGBA+ health research policy partnerships competition

The integration of sex and gender into the health policy process is complex and requires a deep understanding of the scientific literature, including how to apply SGBA+ in a way that is informed by the best available evidence. Recognizing this gap in knowledge, the IGH and Health Canada’s Gender and Health Unit collaborated to fund the SGBA+ Research Policy Partnerships competition in 2017. This funding opportunity aims to support SGBA+ research and facilitate its transfer into health policy development and practice. The total amount available for the SGBA+ Research Policy competition is $150,000 to fund 2 projects per year, with a maximum funding per grant of $75,000. To date, there have been 7 awardees (https://cihr-irsc.gc.ca/e/51192.html).

Other organizations have also provided specific funding for SGBA+ in CV research per se or through other specialties. The HSFC has committed to advancing SGBA+ and improving health for all and requires applicants to integrate SGBA in all research designs (https://www.heartandstroke.ca/what-we-do/research/strategy/funding-excellence). Also, the Women’s Xchange at Women’s College Hospital (https://womensxchange.womensresearch.ca/challenge/previously-funded-projects) has funded more than 140 community-based projects (2013-2019) targeting women’s health issues (hypertension, CAD, inflammatory arthritis) across various sociodemographic and racial-ethnic populations. The Alpha Phi Foundation has provided an annual Heart to Heart grant since 1993, specifically targeted to women’s heart health. This award, based in the United States, is open to Canadian applicants; however, no funding to date has been secured by a Canadian applicant (https://alphaphifoundation.org/vital-programs/womens-heart-health/heart-to-heart-grant). Shared risk factors in CVD with other chronic diseases (eg, cancers, inflammatory diseases) have been evident in funded projects offered by other national charitable organizations. The Canadian Cancer Society offers a diverse portfolio of research grants and awards, including Cancer Survivorship Team grants, Emerging Scholar awards, and a variety of operating grants with specific foci. They recently offered Health Equity Research grants using a social determinants research perspective in identifying and defining health and health equity. Health equity grants include applications that address the broad range of personal, social, and economic factors that determine individual and population health, including sex and gender. One of the mandates of the Arthritis Society Canada’s research program is to promote the best treatments and care for those living with arthritis. They offer a host of funding opportunities that include Ignite Innovation grants, Strategic Operating grants, Stars Career Development awards, Postdoctoral Fellowships, and PhD salary awards (https://arthritis.ca/researchers/competition-results).

### CIHR Planning and Dissemination grants

The CIHR Planning and Dissemination grants aim to support the planning and creation of events that contribute to the advancement of health research. To meet the needs of their respective communities and support their mandates, each of the participating CIHR institutes has tailored the specific requirements of this funding opportunity. Specifically, the IGH currently provides $40,000 to fund 2 Planning and Dissemination grants focused on facilitating the uptake of sex and gender considerations into health research and policy. The event must take place in Canada and the presenter list must reflect diversity and gender parity. In addition, the nominated Principal Applicant must have completed 1 of the sex- and gender-based analysis online training modules through the CIHR IGH and submit a Certificate of Completion. Overall, the success of this funding opportunity will increase the number of knowledge translation events that focus on SGBA+ in health research.

### Sex and gender research chairs

As the field of sex and gender science continues to grow, CIHR recognized the need for discipline-specific chairs to increase visibility and promote innovation. The Sex and Gender Science Chairs funding opportunity aims to support research into the sex- and gender-related mechanisms that underlie risk, incidence, and response of various disease processes. The maximum amount of funds available per chair is $175,000 per year for a total of 4 years. To be eligible for this opportunity, researchers must have completed 1 of the sex- and gender-based analysis online training modules developed by the CIHR IGH and submit a Certificate of Completion (https://cihr-irsc.gc.ca/e/51596.html).

The HSFC has been instrumental in providing funding to advance knowledge in women’s CV health, funding chair and professorship positions for early and midcareer investigators, early career chair positions for Indigenous women’s heart and brain health, and most recently IMPACT awards linking heart and brain health (including heart disease, stroke, and vascular cognitive impairment).

### Women’s Health Clinical Mentorship Grant

The Women’s Health Clinical Mentorship Grant was developed to advance women’s health research and provide mentorship for trainees interested in improving clinical care for women. Each trainee/mentor pair can apply for funding for a 1-year research project. This funding opportunity includes $600,000 total funding for 12 mentorship grants ($50,000 each). To be eligible for this grant, both applicants must have successfully completed 1 of the sex- and gender-based analysis training modules developed by the CIHR IGH and submit a Certificate of Completion. This award opportunity has had considerable uptake in the field of CV research since its inception in 2019. It is expected that the Women’s Health Clinical Mentorship Grant will continue to position Canadian researchers at the forefront of women’s health studies (https://cihr-irsc.gc.ca/e/51599.html).

### Indigenous gender and wellness initiative

The importance of gender is often overlooked in Indigenous health and wellness. The Indigenous Gender and Wellness (IGW) Initiative is a 3-phase program that aims to improve health and wellness for Indigenous peoples from a gendered perspective. Implemented in 2019, the first phase of this initiative distributed travel awards to bring interested Indigenous peoples and researchers to attend an idea fair and learning circle on Indigenous gender and wellness. The event facilitated collaboration between Indigenous peoples and allies and lent itself to the second phase of this initiative, the Indigenous Gender and Wellness Development grants. These grants are intended to support idea fair attendees to continue working on their projects. This funding opportunity offered $1,680,000 to support > 22 grants up to $75,000. The IGW Team grant funding opportunity is the third phase of the IGW Initiative and provides funding of $7,000,000 for 14 grants to be awarded to Indigenous-led projects relating to gender and holistic health (https://cihr-irsc.gc.ca/e/51988.html).

### Sex as a Biological Variable Supplement: COVID-19

In the midst of the current COVID-19 pandemic, the Sex as a Biological Variable Supplement: COVID-19 opportunity provides supplementary funds to PIs awarded a Canadian 2019 Novel Coronavirus Rapid Research Operating Grant. A maximum of $50,000 can be requested over 1 year. The purpose of this funding opportunity is to uncover mechanistic explanations of sex differences observed in COVID-19 severity (https://cihr-irsc.gc.ca/e/52009.html).

## Journal Publications

***Reporting results disaggregated according to sex/gender should be mandatory for all published articles. Indeed sex- and gender-specific reporting of study results is required to understand the therapeutic efficacy and side effects of pharmacologic, device, and lifestyle interventions for women and men, as well as the understanding of unique physiologic responses. Sex- and gender-specific findings in a published evidence base is required to enable the development of appropriate recommendations in CV guideline documents, which currently are sorely lacking.***

Publication of research findings reporting sex- or gender-specific experiences of cardiac health outcomes are an important component of understanding women’s experiences of CVD. Specific to women during pregnancy, the Canadian Cardiovascular Society (CCS) recently published a clinical practice update on the management of CVD for pregnant patients.^47^ This update provides recommendations on the use of medications along with preconception and pregnancy counselling for various CVDs, including arrhythmias, hypertension, cardiomyopathies, valvular heart disease, congenital heart disease, and pulmonary hypertension during pregnancy. Most of the past CCS guidelines or consensus documents, apart from those focused on pregnancy-related conditions, have not reported sex-specific recommendations because of a lack of published data. The guidelines on antiplatelet therapy in CAD attempted to evaluate sex-based data and showed extensive reporting gaps such that formal recommendations could not be made. There is a concerted effort by the CCS Guidelines Committee asking that such data be identified and where available and feasible, to be used to formulate sex-specific recommendations.

### Sex and gender equity in journal publications

Since 1991, when Dr Bernadette Healey coined the term “Yentl Syndrome” to underscore the lack of diagnosis and treatment of CVD in women,^48^ there have been repeated calls for sex-specific research, reporting, and guidelines for CVD.^48^, ^49^, ^50^, ^51^, ^52^, ^53^, ^54^ Despite these regular calls for understanding women-specific experiences of CVD, advances in research and reporting of women-specific experiences remain low.^51^ Further, sex-specific data reporting remains low among randomized clinical trials of pharmacological interventions for CVD.^55^

The importance of sex-specific reporting of CVD research has been highlighted in many international society statements, including the European Society for Cardiology,^52^ American Heart Association,^53^ and the CCS,^56^ specifically calling for sex-specific reporting of clinical trial findings. In response to a critical deficiency in reporting sex- and gender-disaggregating data, the SAGER guidelines were developed. From applications for grants to the development of publications, this methodological framework developed by the European Association of Science Editors can be applied to all time points of the scientific process.^57^, ^58^, ^59^ Accordingly, the SAGER guidelines have now been translated into 6 languages and their use is encouraged by large funding agencies such as the CIHR.^59^ The endorsement of these guidelines by journal publications supports identifying and recognizing sex-specific experiences. However, none of the endorsing organizations of these guidelines are cardiology journals.

A recent bibliometric analysis of all CV publications, including Canada and international, identified only 13% of more than 189,000 publications from 2006-2015 reporting experiences specific to women or female participants.^51^ Although there has been a small increase (3.4%) in the proportion of research articles reporting women-specific experiences, there has not been an increase in the proportion of clinical trials, meta analyses, or review articles reporting women-specific experiences.^51^^,^^60^ Further, a review of randomized controlled trials of pharmacological interventions for CVD has not shown an increase in sex-specific reporting of results since guidelines calling for sex-specific reporting have been published.^55^

Although recognition of the importance of researching, identifying, and reporting women-specific experiences of and treatments for CVD have been recognized, the proportion of published research evaluating and reporting women-specific experiences continues to remain low. With increased awareness and understanding of the unique experiences of CVD among women and the risks/consequences of overlooking these sex and gender differences, the inclusion of women and sex-specific CVD research remains a high priority.

### Guideline publication

The lack of progress in researching, identifying, and reporting women-specific experiences of and treatments for CVD still limits the available evidence to support guidelines and recommendations specific to the treatment and management of CVD for women. This lack of knowledge is evident in the lack of a comprehensive women-specific guideline on CVD in Canada, with infrequent and inconsistent comments in existing guidelines addressing women as a “special population.”^7^ Within guidelines for familial hypercholesterolemia, the only guidelines specific to women are pregnancy-related.^61^ Canadian guidelines around antiplatelet therapy do not mention women or any specific similarities or differences in experiences between men and women.^62^ Guidelines for anemia, biomarkers, and therapeutic trial implications also do not include any specific recommendations or guidelines for women.^63^ Guidelines for valvular heart diseases introduced the sex-specific thresholds for identifying severe aortic valve calcification although they did not provide any other women-specific recommendation,^64^ except for concomitant or expected pregnancy. Overall, a greater understanding of women’s specific experiences and treatments of CVD are needed.

### Publication on CVD and pregnancy

Among women in Canada who have heart disease, pregnancy is associated with significant morbidity.^47^^,^^65^ Women in Canada who experience hypertensive disorders during pregnancy, including preeclampsia and pregnancy-induced hypertension, are at high risk for premature CVD, with markedly reduced 30-year survival rates and vascular events beginning at an average age of 38 years.^66^^,^^67^ Sufficient publications on the experiences of hypertension during pregnancy among women (almost only non-Hispanic white women) in Canada are available to support guidelines for management of hypertension during pregnancy.^67^^,^^68^ Within Canada, the incidence of myocardial infarction during pregnancy is 1.15/100,000 pregnancies, well below experiences in the United States, as well as low rates of maternal mortality of 0.06/100,000 pregnancies.^69^ Numerous adverse events of pregnancy have been linked to increased hemorrhage with blood transfusion and other severe maternal morbidities.^70^ Severe to mild events during pregnancy, such as preterm birth, stillbirth, placental abruption, gestational diabetes, low birth weight, etc, are risk factors for future CVD. Further, a woman’s future risk of CVD after adverse pregnancy events is affected by socioeconomic status, race, and ethnicity, highlighting the importance of evaluating diverse women’s experiences.^70^ Assessments of oral contraception risks of myocardial infarction and cerebrovascular accidents have also been assessed in Canada.^71^

### Publication on women and myocardial infarction

Canadian guidelines on ST-elevation myocardial infarction acknowledge the need for more research and publications regarding the experiences of women.^72^ Similarly, guidelines for ischemic heart disease do not include any specific recommendations for women.^73^ Canadian guidelines on revascularization of multivessel CAD also lack any specific recommendations or guidelines for women.^74^ These guidelines highlight this significant gap in understanding and treatment of myocardial infarction for women and a clear need for more research and publications on women’s specific experiences and treatment of CVD.

### Publication on women and atrial fibrillation

The CCS guidelines for management of atrial fibrillation include recognition of the importance of sex, with greater age-adjusted incidence among men, but greater numbers of female patients.^75^ Literature around women’s experiences of atrial fibrillation have identified differences in presentation, effects, mortality, and stroke experiences.^75^ Available research also identifies inappropriate underutilization or underdosing of medications for women experiencing atrial fibrillation or with associated complications and poorer outcomes.^75^ Although guidelines on the assessment and management of syncope recognize greater incidence and prevalence among women, sex-specific recommendations are not available. Additional research is required to further identify experiences, complications, and treatments of arrhythmias specific to women.

### Publication on women and heart failure

Evidence around women’s experiences of heart failure in Canada is limited. Guidelines from the CCS for a pharmacological standard of care for heart failure do not consider women-specific experiences.^76^ In recent clinical trials, sex differences in benefits of some heart failure treatments have been recognized,^8^^,^^77^ though limited sex-specific recommendations have yet to emerge.^1^^,^^4^^,^^76^^,^^78^ Position statements on the evaluation and management of patients with cardiac amyloidosis do not address women-specific experiences or treatments, likely due to a lack of available evidence.^79^ Specific recommendations and guidelines around heart failure specific to women in Canada are limited to pregnancy-related recommendations and guidelines.^80^

### Publication on women, cancer, and CVD

The CCS guidelines around complications of cancer therapy identify women as being at high risk of asymptomatic left ventricular dysfunction and anthracycline-induced heart failure.^81^ However, there are no Canadian guidelines or recommendations for CV complications of cancer specific to women.^81^

### Publication on women and valvular heart diseases

Several publications have reported sex specificity in valvular heart disease,^82^^,^^83^ especially in aortic stenosis^17^^,^^84^, ^85^, ^86^ and mitral regurgitation.^87^^,^^88^ These articles report many differences in pathophysiology, diagnosis, management, and/or outcomes; however, guidelines provide only 1 sex-specific recommendation, which is the sex-specific thresholds for identification of severe aortic stenosis using calcification measured using computed tomography.^64^ Nevertheless, the Heart Valve Voice Canada highlighted in their patient journey report that “the patient’s care journey is often different for women.”^89^

## Authors, Editorial Boards, and Reviewers

***Studies across several published sciences, including medicine, showed that women are less likely than men to be first or last authors, members of the editorial boards, or reviewers especially in high impact factor journals. This under-representation might partly explain the shortage of women at the top ranks of academic positions.***

To enhance the reporting of sex-based findings and experiences, it is important first to understand the current state of affairs when it comes to the vast under-representation of women across the CV sciences, surgery, and medicine within Canada.

The necessity of publication in research is well known and is often summarized as “publish or perish.” In addition, the coauthor position is important, especially first author for trainees and last/senior author for PIs. The representation of women as scientific authors in cardiology has historically been low.^90^ Despite an increase in female authorship,^90^^,^^91^ there is still a deficit of women as first and last authors, especially in high impact journals, in original research and editorials. Women are also under-represented as leaders of CV randomized controlled trials, representing 1 in 10 lead authors of CV trials published in high impact journals.^11^ Some publications tried to explain why women were less productive (ie, greater family responsibility, less welcoming work environment, etc), but recently it was suggested that this is not a matter of productivity, but rather, that their work is undervalued.^92^

This lack of women as leading authors in publication is also reflected in the few female authors in most of the cardiology guidelines. Interestingly, having a female as the senior author in cardiology publications can improve the quality, visibility, and sex/gender sensitivity of the research.^93^^,^^94^

The *Canadian Journal of Cardiology* (CJC), *CJC Open*, and *CJC Pediatric & Congenital Heart Disease* are the main CV journals espoused by the CCS. Although past editorial board membership has been largely men, there has been an increase over time in the number of women (CJC 11%, *CJC Open* 37%) and women trainees (45%) as members of the editorial board and mentorship program, respectively.^95^ *CJC Open* is also being led by a female editor-in-chief since its inception in 2019. In comparison, cardiac surgery journals report 10% of female representation collectively on their editorial boards, ranging from 0 to 30% for an individual journal.^96^ The statistically significant positive correlation identified between percentage of women on cardiac surgery journal editorial boards and the journal impact factor (*r* = 0.8; *P* < 0.001) underscores the value of this diversity.^96^

Female representation in reviewers’ teams and editorial boards of cardiology journals is low, but there have been efforts to increase gender diversity in recent years. Recent studies have shown that the proportion of female editors-in-chief increased from 0% in 2005 to 20% in 2015 or 21% in 2019 among more than 20 cardiology journals.^97^^,^^98^ However, there is still need for continued improvement, because less than 25% of editorial board members are female.^97^^,^^98^

Although there has been progress in increasing gender diversity in editorial boards of cardiology journals, there is still work to be done to address sex disparities and promote equity in leadership roles. Increasing female representation in editorial boards can help to promote diversity of perspectives and ensure that the needs of all members of the scientific community are represented.

## Meetings and Conferences

***Women’s heart health research is less likely to be presented at conferences, or relegated to categories described as a subset of “special populations,” which can perpetuate the knowledge gap in women with heart disease. Addressing this issue requires increasing the representation of women’s heart health topics in scientific conferences and providing more opportunities for women scientists to lead investigative trials and present their work.***

Within Canada, the CV community convenes at the Canadian Cardiovascular Congress (CCC) on an annual basis. The CCS recently forged a “One Heart Team” model of partnership with its affiliates that represent cardiac surgery and other disciplines (eg, cardiac imaging and echocardiography, critical care cardiology, adult congenital cardiology, heart failure and transplant cardiology, interventional cardiology, pediatric cardiology, etc). Each of these affiliates are represented at the CCC, but have their own annual meetings or events. Although many journals and funding agencies are beginning to use the SAGER framework to encourage reporting of sex-based data, there is no formal requirement to use this for research or symposia presentations at the CCC or CCS affiliate meetings. Over the past 2 years, however, the CCC has incorporated a dedicated research session and award within their programming in which trainees present sex-based research studies. A similar trend has been variably incorporated within the CCS affiliate meetings.

The only environmental scan of the annual CCC meetings highlighted several interesting trends around female representation including^95^: (1) few women (n = 4) served as Scientific Program Committee chairs, and only after 2016; and (2) 30% or fewer women were members of the Scientific Program Committee (23%) or served as major symposia panelists (30%) at the CCC between 2014 and 2018. Importantly, with a targeted effort to solicit diversity including 50% women through workshop proposal submissions, there has been an increase in the number of women as planning committee members, session chairs, and speakers. An analysis of the Canadian Association of Thoracic Surgeons annual meetings from 1997 to 2020 revealed that most lecturers (88%) and invited speakers (67%) were white men.^99^

The Canadian Women’s Heart Health Summit was launched in 2016 and is cohosted by the University of Ottawa Heart Institute and HSFC. The Canadian Women’s Heart Health Summit is a biannual, multiday conference that focuses exclusively on sex- and gender-specific evidence related to heart, brain, and vascular health. Its objectives are to: (1) bring together national and international leaders, knowledge users, and women with lived experience to develop and disseminate strategies to improve heart, brain, and vascular health among women; (2) identify emerging areas of research and clinical practice related to heart, brain, and vascular conditions affecting women across their life span; (3) address gaps in research and clinical practice for heart, brain, and vascular disease in women, improving risk stratification, diagnosis, and therapy from a sex and gender perspective; and, (4) promote networking among clinicians, scientists, policy-makers, trainees, and women with lived experience, facilitating translation of knowledge that will ultimately improve the health of women.

The Canadian Organization for Gender and Sex (COGS) research was launched in 2019 and had its first meeting in 2020. The COGS mission is to forge a transformative, transdisciplinary field of sex and gender science. Its objectives are to: integrate sex and gender in research, bolster sex and gender science with transformative new methods, measures, and technologies, promote the transdisciplinary field of sex and gender science, build strong networks among policy makers, service providers, the technology community, funders, and the public to advance sex and gender science, and train the next generation in the field of sex and gender scientists.

These data altogether highlight that editorial boards and conference leadership disparities might lead to slower academic advancement of under-represented groups and fewer publications or leadership roles related to less inclusive review processes or engagement. To address this critical under-representation of women and racialized peoples within the CV community, the Canadian Association of Thoracic Surgeons and the CCS have established equity, diversity, and inclusion task forces and the CJC family of journals has actively sought to enhance opportunities for women. Additional methods to enhance female representation at conferences include the evolving use of social media, promotional media campaigns, and allyship through the formation of women’s alliances to amplify gender equality activism and foster the advancement of women to these opportunities.

## Translation of Research Knowledge into Clinician Training/Knowledge

Effective knowledge translation and accurate guidelines/knowledge application improve patient outcomes and reduce health care costs. It is particularly important to identify and reduce barriers to improve knowledge translation and guideline adherence in women’s heart health.

There is universal agreement that clinical guidelines and patient care should follow the best up to date evidence. Patients rely on health care professionals to provide them with the most recent, transparent, safe, and effective health care for their families and society. However, there is a considerable gap between the care a patient receives and what is recommended by clinical guidelines informed by research evidence.^100^, ^101^, ^102^ The guideline recommendations are followed on average, in 67% of clinical decisions, but there is a significant variation among physicians and the guidelines.^103^

There are various barriers that could lead to the failure of the implementation of clinical guidelines, and some of the barriers are related to the sex and gender of the physician and/or the patient. A meta-analysis on guidelines adherence reveal that patient’s sex is a major predictor of decreased guidelines adherence in CVD. Women experiencing myocardial infarction received fewer coronary angiography examinations, and were less often prescribed β-blockers, angiotensin converting enzyme inhibitors, angiotensin receptor blockers, or lipid-lowering therapy.^104^ Interestingly, the physician’s sex was of less importance, with only a difference in β-blocker prescriptions (higher in female physicians). All of these differences are less important (or absent) in large centres with cardiology departments. The differences in CVD in women might explain a part of these differences in treatment adherence. However, these differences have been described and should be known. Barriers in knowledge transmission appear to also be worse for women (see the paragraphs on journals and conferences), who are also more inclined to report sex-specific results.

Although change might take time, failure to implement and translate research knowledge into practice not only contributes to suboptimal care for patients but also affects health care organizations and society by missing out on the benefits of innovation and potential financial value gained on research investment.^105^ Knowledge translation is not linear and involves many processes, systems, and interactions between all stake holders and knowledge users.

## Recommendations

Despite action to improve understanding of women’s heart health at the federal or provincial levels (Table 4), more action is still required (Table 5). Although the inclusion of women in CV clinical trials has improved, it still remains an important priority, and the inclusion of women who identify as Indigenous and/or racialized is a key demographic. We should add ethnic/ethnicity and Indigenous to all of the sex and gender approaches in this section to be genuinely inclusive as authors of this publication.

**Table 4:** Current actions (2020) on women’s health and SGBA by federal and provincial ministries

	Current action on women/SGBA/GBA+	Source
**Federal ministries**		
Health Canada	Health Canada is committed to incorporating sex and gender considerations into all research, legislation, policies, regulations, and programs. Health Canada will integrate SGBA into Health Canada’s practices by building department capacity, strengthening the evidence base by providing expertise, and increasing accountability. Maintain research partnerships between CIHR IGH, the CIHR Institute of Aboriginal People’s Health, and the Health Canada Gender and Health Unit to bridge gaps in research knowledge and policy development. Additionally, Health Canada will provide expert-level advice on gender research into the following areas: home care, cannabis, vaping, health product labelling, pest management, health products, healthy child development, and workplace health.	https://www.canada.ca/en/health-canada/corporate/transparency/corporate-management-reporting/health-portfolio-sex-gender-based-analysis-action-plan.html
Innovation, Science and Economic Development Canada	Introduced a new Women Entrepreneurship Strategy to better support the growth of women entrepreneurs. Aim to strengthen Canada’s scientific research to reflect the diversity in Canada—including support for women, Indigenous, and minorities. Support early-career researchers including increasing the number of women nominated for Canada Research Chairs.	https://www.ic.gc.ca/eic/site/017.nsf/vwapj/ISED_2018-19_Departmental_Plan-eng.pdf/$FILE/ISED_2018-19_Departmental_Plan-eng.pdf
PHAC	PHAC is committed to enhancing GBA+ through training and the building of capacity, and by increasing accountability, monitoring, and reporting. PHAC will focus on integration of sex- and gender-based analysis into surveillance activities, science, policy, programs, and evaluation. Pan-Canadian Health Inequalities Reporting Initiative: strengthen data and infrastructure reporting. Included in this collaboration are Statistics Canada, and the Canadian Institute of Health Information.	https://www.canada.ca/en/public-health.html
NRC	NRC is in support of fostering STEM careers among women through mentorship and networking.	• https://www.canada.ca/en/national-research-council/news/2019/02/empowering-women-in-chemistry-through-mentorship-and-networking.html • https://www.nrc-cnrc.gc.ca/eng/reports/2018_2019/dp_2018_2019/gba.html
Statistics Canada	Statistics Canada to collect more inclusive data on sex and gender, and release new variables and classifications for sex and gender. Statistics Canada created the new Gender, Diversity and Inclusion Statistics Hub in 2018.	• https://www150.statcan.gc.ca/n1/daily-quotidien/180413/dq180413e-eng.htm • https://www150.statcan.gc.ca/n1/daily-quotidien/180926/dq180926c-eng.htm
Department for Women and Gender Equality (formerly Status of Women Canada)	The Department for Women and Gender Equality focuses on building equality in 3 key areas: 1. Economic security; 2. Leadership; and 3. Gender-based violence. Also play a role in government-wide implementation of GBA+. As part of the Gender Results Framework, there is movement to improve diversity in research by increasing female representation on research teams.	• https://cfc-swc.gc.ca/ • https://cfc-swc.gc.ca/trans/account-resp/pr/dp-pm/1920/dp-pm-en.pdf
**Federal granting agencies**		
CIHR	There are 3 components to the CIHR GBA+ Framework, which include: 1. GBA+ in CIHR-funded research: ensures that GBA+ is taken into account in research design, methods, analysis, interpretation, and dissemination of findings; 2. GBA+ in CIHR’s funding system: ensures equitable access to CIHR funding across eligible individuals; and 3. GBA+ in CIHR’s workplace: ensures CIHR conducts business in an equitable manner and takes into account the effect of business decisions on diverse groups. CIHR also sponsors sex- and gender-based health research grants across universities in Canada. CIHR has also established the IGH to focus on sex and gender influences on health. The IGH strategic plan (2018-2023) is guided by 3 core strategic directions: 1. Transforming health research to foster the integration of sex and gender in science; 2. Promoting innovative methods (new research and approaches) in the field of sex and gender; and 3. Transforming health outcome by ensuring knowledge is translated into improved health. The IGH has been involved in the following activities: • Sex and gender champions on team grants • Launch of 3 training modules and mandatory training • Helped to develop and adopt SAGER guidelines • Knowledge translation through webinars, presentations, interviews, and multi-institute partnerships • CIHR develops sex and gender analysis in research action plan (2017-2019) The IGH is involved in international knowledge exchange and has been invited to present numerous summits including the Gender Medicine Summit (Italy), Gender and Health Congress (The Netherlands), and the Sex and Gender Health Education Summit (United States). To support their strategic plan, IGH will: • Assist researchers to apply SGBA+ as applicants and peer reviewers • Build capacity by targeting trainees and new investigators as early adopters • Work with research ethics board to ensure ethical considerations regarding sex and gender are implemented in research • Encourage journal editors to implement best practices for reporting of sex and gender in peer-reviewed publications • Guide and support CIHR in their implementation of SGBA+ action plan • Engage in activities to raise awareness • Promote SAGER guidelines among Canadian health science and medical journals	• http://www.cihr-irsc.gc.ca/e/50970.html • http://www.cihr-irsc.gc.ca/e/documents/igh_strategic_plan_2018-2023-e.pdf
NSERC	NSERC and CIHR are 2 of 16 domestic and international partners in GENDER-NET Plus. GENDER-NET Plus mission is to fund research projects that promote the integration of sex and gender analysis into research at international levels. The research topic of sex, gender, and health is one of the main areas of focus. In addition to GENDER-NET Plus, NSERC is committed to making GBA+ a key component of research excellence. Research proposals must discuss the balance of men and women on research teams, and analyze the possible differences between women and men, boys and girls, and male and female participants in the research and innovation content of the proposed project. Additionally, gender and sex ratios in research designs should have the potential to enhance scientific quality and usefulness of proposed research. Spearheading the Made-in-Canada Athena SWAN initiative, which has recently released their draft Charter in February of 2019.	• http://www.nserc-crsng.gc.ca/Professors-Professeurs/Grants-Subs/GenderNet-GenderNet_eng.asp • http://www.nserc-crsng.gc.ca/_doc/EDI/EDIpresentation_EN.pdf • http://gender-net-plus.eu/wp-content/uploads/2017/11/GENDER-NET-Plus-Further-Requirements-1.pdf
SSHRC	As part of its EDI for Research Excellence Implementation Plan, SSHRC has, in 2018-2019, developed an approach to systematically include GBA+ in program evaluations. SSHRC continue working with the other granting agencies to develop plans to achieve greater diversity among research funding recipients, including improved support for women, under-represented groups, and early-career researchers. In addition, Budget 2018 has proposed a new investment for the Canada Research Chairs Program to better support early-career researchers, while increasing diversity among nominated researchers, including increasing the number of women who are nominated for Canada Research Chairs.	http://www.sshrc-crsh.gc.ca/about-au_sujet/publications/dp/2018-2019/2018-2019_DP_FinalE.pdf
**Federal committees**		
Canada Research Coordinating Committee	Established by the Minister of Science and Sport and Minister of Health in October 2017. The committee was founded to address 5 key issues including EDI in research. The committee brings together the executive heads of Canada’s 3 funding agencies (CIHR, NSERC, SSHRC) and the Canada Foundation for Innovation, Health Canada, Science and Economic Development Canada, and the NRC. Regarding EDI the committee has: • Developed a triagency EDI Action Plan (2018) • Provided mandatory GBA+ training for all triagency staff • Launched a pilot EDI Institutional Capacity Building Grant program (2018) • Launched a draft Made-in-Canada Athena SWAN Charter (2019)	http://www.sshrc-crsh.gc.ca/CRCC-CCRC/pdf/SSHRC_CRCC_progress_report_2019-eng.pdf
**Provincial ministries**		
Ontario		
Ministry of Health and Long-Term Care	All HSRF-funded programs include SGBA where applicable. The HSRF was created to promote research in complex health issues in Ontario. Through the HSRF, women’s health research has been integrated into policy-relevant health services provided by the ministry.	http://www.health.gov.on.ca/en/pro/ministry/research/hsrf_faq.aspx#general
Ministry of Labour	Pay Equity Act ensures that women and men earn equal pay for work of equal value. Occupational Health and Safety Act definition of workplace harassment includes harassment on the basis of sex and gender.	https://www.ontario.ca/document/your-guide-employment-standards-act-0/equal-pay-equal-work#section-2
British Columbia		
Ministry of Health	In 2008, a report was released on a provincial women’s health strategy: • To advance knowledge on women’s health issues and to ensure women receive gender-sensitive care • To understand the contributions of sex and gender to health conditions through the incorporation of a sex and gendered lens in research • To offer gender-inclusive health training	http://bccewh.bc.ca/wp-content/uploads/2012/05/2008_Further-Advancing-the-Health-of-Girls-and-Women.pdf
Ministry of Mental Health and Addictions	Unclear if steps are being taken to address SGBA+ in mental health and addictions.	
Alberta		
Ministry of Health	Cooperate with the Status of Women (Alberta) to incorporate GBA+ into the Ministry of Health.	
Status of Women (Alberta)	Status of Women works with Alberta government ministries to apply GBA+ to policies, programs, and legislation across the government. Main areas of focus are: • Women’s economic security • Gender-based violence • Women in leadership Following CIHR’s framework of incorporating sex- and gender-based analysis into research grant approvals.	https://www.alberta.ca/gender-based-analysis.aspx
Manitoba		
Health, Seniors and Active Living	SGBA/GBA+ N/A	
Nova Scotia		
Department of Health and Wellness	SGBA/GBA+ N/A	
Nova Scotia Advisory Council on the Status of Women	Concerned with all areas of women’s life including: family life, health, education, legal rights, and experiences of violence. The advisory council recognizes the work performed by the Department for Women and Gender Equality on GBA+.	https://women.novascotia.ca/about-status-women/advisory-council
New Brunswick		
Department of Health	Recognizes health inequalities between men and women.	https://www2.gnb.ca/content/dam/gnb/Departments/h-s/pdf/en/Publications/HealthInequitiesNewBrunswick2016.pdf
Department of Women’s Equality	The Department of Women’s Equality has a mandate to promote gender equality and reduce systematic discrimination. The department aims to provide support and coordinate efforts with other provincial ministries in areas concerning women’s person, economic, and social security.	https://www2.gnb.ca/content/gnb/en/contacts/dept_renderer.153.html#mandates
Yukon		
Health and Social Services	SGBA/GBA+ N/A	
Women’s Directorate	The women’s directorate focuses on promoting gender equitable outcomes by: • Providing policy development and research within government legislations • Targeting public education • Supporting community initiatives that advance female equality	https://yukon.ca/sites/yukon.ca/files/fin/fin-budget2018-womens-directorate.pdf
Northwest Territories		
Health and Social Services	SGBA/GBA+ N/A	

CIHR, Canadian Institutes of Health Research; EDI, equity, diversity, and inclusion; GBA+, Gender-Based Analysis Plus; HSRF, Health System Research Fund; IGH, Institute of Gender and Health; N/A, not applicable; NRC, National Research Council; NSERC, Natural Sciences and Engineering Research Council of Canada; PHAC, Public Health Agency of Canada; SAGER, Sex And Gender Equity in Research; SGBA, sex and gender-based analysis; SSHRC, Social Sciences and Humanities Research Council; STEM, sciences, technology, engineering and mathematics; SWAN, Scientific Women’s Academic Network.

**Table 5:** Call for action—recommendations

Recommended action	Justification and anticipated outcomes
**Trainee (education)**	
Emphasize the importance of sex and gender considerations in research	Research trainees should understand that sex and gender play a crucial role in shaping health outcomes and that ignoring these factors can lead to incomplete or inaccurate conclusions
Provide training on sex and gender concepts and methods	Trainees should receive training on the differences between sex and gender, how to incorporate these concepts into research design and analysis, and how to collect and analyze data that accounts for sex and gender
Encourage the use of SGBA+ tools	Trainees should be provided with tools such as SGBA+ checklists to ensure they have considered these factors in all aspects of their research
Facilitate collaboration with sex and gender experts	Trainees should be encouraged to collaborate with experts in sex and gender research, either within their own institution or through external partnerships, to improve the quality of their research
Foster a culture of inclusivity and diversity	Trainees should be taught to respect and value diverse perspectives, and to consider how social, cultural, and historical factors influence sex and gender dynamics in research
**Faculty/scientists/researchers**	
Foster a culture of inclusivity and diversity	Faculty, scientists, and researchers should actively work to promote a culture that values diverse perspectives and recognizes the importance of considering social, cultural, and historical factors that influence sex and gender dynamics in research
Provide ongoing education and training on sex and gender concepts and methods	Faculty, scientists, and researchers should provide ongoing training to their colleagues and trainees on how to incorporate sex and gender considerations into research design, analysis, and reporting. This can include workshops, seminars, and other educational opportunities
Encourage interdisciplinary collaboration	Faculty, scientists, and researchers should encourage collaboration across disciplines and with experts in sex and gender research to improve the quality of their research
Use SGBA+ tools and resources	Faculty, scientists, and researchers should use SGBA+ checklists and other resources to ensure that they have considered these factors in all aspects of their research
Publish and disseminate research findings that account for sex and gender	Faculty, scientists, and researchers should make a concerted effort to publish research findings that account for sex and gender, and to disseminate this information to the broader scientific community and to the public
**Funding agencies**	
Require SGBA+ considerations in all grant applications	Implement policy change to require the integration of sex and gender into all funding applications
Provide funding opportunities specifically for sex and gender research	Create funding opportunities for research projects which specifically address sex- and gender-based research
Encourage interdisciplinary collaboration across fields and disciplines	Improve the quality and comprehensiveness of sex and gender research
Foster a culture of inclusivity and diversity	Promote a culture that values diverse perspectives and recognizes the importance of considering social, cultural, and historical factors that influence sex and gender dynamics in research
Monitor and evaluate the integration of sex and gender considerations in research projects they fund	Provide feedback and support to researchers as needed to assure successful incorporation of SGBA+ into results and conclusions
**Journals, societies, and conferences**	
Provide guidance to authors on how to incorporate sex and gender considerations into their research	The use of SGBA+ should be included in checklists and other resources
Actively encourage the publication of research that incorporates sex and gender considerations	The importance of this research should be highlighted to their readership to improve awareness and knowledge translation
Require the use of appropriate sex and gender terminology in all research reporting	Standardization and familiarization of SGBA+ terminology will ensure clarity and accuracy
Evaluate the integration of sex and gender considerations in all submitted manuscripts/abstracts	Provide feedback to authors as needed to achieve this goal
Promote a culture that values diverse perspectives	Elevate the importance of social, cultural, and historical considerations that influence sex and gender dynamics in research
**Patients/public**	
Seek education	Become aware of the importance of sex and gender considerations in research and how these factors can affect health outcomes
Engage with researchers, health care providers, and policymakers	Communicate the importance of these factors
Participate in sex and gender research studies	Contribute directly, enabling a more comprehensive understanding of how these factors affect health outcomes
Share their experiences and perspectives	Shared experiences and perspectives of patient partners on how sex and gender affected their health and health care experiences will help inform and guide future research and policy decisions

SGBA+, Sex- and Gender-Based Analysis Plus.

### Trainees (education)

To improve the inclusion of sex- and gender-based analyses in CVD research, improving the education of trainees is essential. The 5 major points to implement would be:

• Emphasize the importance of sex and gender considerations in CVD research.
• Provide training on sex and gender concepts and methods.
• Encourage the use of sex and gender analysis tools.
• Facilitate collaboration with sex and gender CVD experts.
• Foster a culture of inclusivity, diversity, equity, and accessibility to encourage trainees to maintain this culture in their future career which will likely involve mentorship.

### Faculty/scientists/researchers

Faculty, scientists, and researchers must recognize that sex and gender play a critical role in shaping CVD health outcomes and that incorporating these factors into research can lead to more accurate and comprehensive results. They thus should (Table 5):

• Foster a culture of inclusivity, diversity, equality, and accessibility that applies to their studies and research training environment.
• Prioritize education and training on sex and gender concepts in women’s heart health and methods for trainees and staff.
• Encourage interdisciplinary collaboration.
• Use sex and gender analysis tools and resources.
• Publish and disseminate research findings that account for sex and gender in women’s heart health.

### Funding agencies

Most federal funding agencies have developed guidelines and policies that require researchers to consider sex and gender factors in their research design, analysis, and reporting. These guidelines must also be used in provincial and local funding agencies. In addition, the funding agency should (Table 5):

• Provide targeted funding opportunities for sex and gender CVD research.
• Encourage interdisciplinary collaboration across fields and disciplines.
• Foster a culture of inclusivity, diversity, equality, and accessibility.
• Monitor and evaluate the integration of sex and gender considerations in CVD research projects they fund.

### Journal and conferences

Scientific journals, learned societies, and scientific conferences should develop and enforce editorial policies that require the reporting of sex and gender considerations. Every clinical trial, animal model, or basic study should be obligated to report results disaggregated according to sex. Thus, journals, societies, and conferences should (Table 5):

• Provide guidance to authors on how to incorporate sex and gender considerations into their CVD research.
• Actively encourage the publication of CVD research that incorporates sex and gender considerations.
• Require the use of appropriate sex and gender terminology in all CVD research reporting to ensure clarity and accuracy.
• Evaluate the integration of sex and gender considerations in all submitted CVD manuscripts and abstracts.
• Promote a culture that values diverse perspectives.

### Patients and public

The patients and public should advocate for the incorporation of sex and gender considerations in CVD research. Thus, they need to (Table 5):

• Educate themselves on the importance of sex and gender considerations in CVD research
• Engage with CVD researchers, health care providers, and policy-makers
• Participate in sex and gender CVD research studies
• Share their experiences and perspectives.

## Conclusions

There are major gaps in knowledge regarding the epidemiology, presentation, diagnosis, management, and outcomes in women’s CVD. Women have unique risk factors for CVD that are often not recognized or addressed by health care providers, leading to poorer health outcomes compared with men. Additionally, there is a lack of sex- and gender-based analysis in research studies, which hinders our understanding of the underlying mechanisms of CVD in women. Addressing these gaps of knowledge requires a multifaceted approach, including improving medical education and training on women’s CV health, increasing sex- and gender-based analysis in research studies, and promoting a culture of inclusivity, diversity, equality, and accessibility in health care. The call to action to improve sex- and gender-based analyses should emphasize the importance of integrating sex and gender considerations into all aspects of research design, execution, and reporting, and provide the necessary support and resources to achieve this goal. By doing so, the research community can contribute to a more equitable and effective research environment that benefits everyone.

## Resources

**CWHHA Web site:**

• English: https://cwhhc.ottawaheart.ca/national-alliance/cwhha
• Français: https://cwhhc.ottawaheart.ca/fr/lalliance-canadienne

**COGS Web site:** https://cogsresearch.ca

**CWHHA Atlas:** https://cwhhc.ottawaheart.ca/national-alliance/projects-and-initiatives/cwhha-atlas

**CWHHA training modules:**

• English: https://cwhhc.ottawaheart.ca/national-alliance/projects-and-initiatives/canadian-womens-heart-health-education-course
• Français: https://cwhhc.ottawaheart.ca/fr/lalliance-canadienne/projets-et-initiatives/initiative-nationale-de-sensibilisation-la-sante-cardiaque-des-femmes

**SAGER guidelines:** https://ease.org.uk/communities/gender-policy-committee/the-sager-guidelines

**CIHR training modules:**

• English: https://cihr-irsc.gc.ca/e/49347.html
• Français: https://cihr-irsc.gc.ca/f/49347.html

**CIHR: How to integrate sex and gender into research:**

• English: https://cihr-irsc.gc.ca/e/50836.html
• Français: https://cihr-irsc.gc.ca/f/50836.html

**HSFC – Women:**

• English: https://www.heartandstroke.ca/women
• Français: https://www.coeuretavc.ca/femmes

**Statistics Canada. Census Profile, 2021 Census of Population:** https://www12.statcan.gc.ca/census-recensement/2021/dp-pd/prof/index.cfm?Lang%20=%20E
